# Supplementary material for: Detecting material state changes in the nucleolus by label-free digital holographic microscopy
Source: EMBO Rep. 2024 Apr 23;25(6):2786–811. doi: 10.1038/s44319-024-00134-5 (PMC11169520; doi:10.1038/s44319-024-00134-5)
Supplement: Supplementary file 1 — Appendix [file 44319_2024_134_MOESM1_ESM.pdf]

Appendix

Table of Content:

Pages 1-2: **Appendix Figure S1**: Image processing pipeline

Pages 3-4: **Appendix Figure S2**: Benchmark of data analysis

Pages 5-6: **Appendix Figure S3**: Comparison of segmentation of the GFP signal by tresholding and of the DHM signal by deep learning

Appendix Figure S1

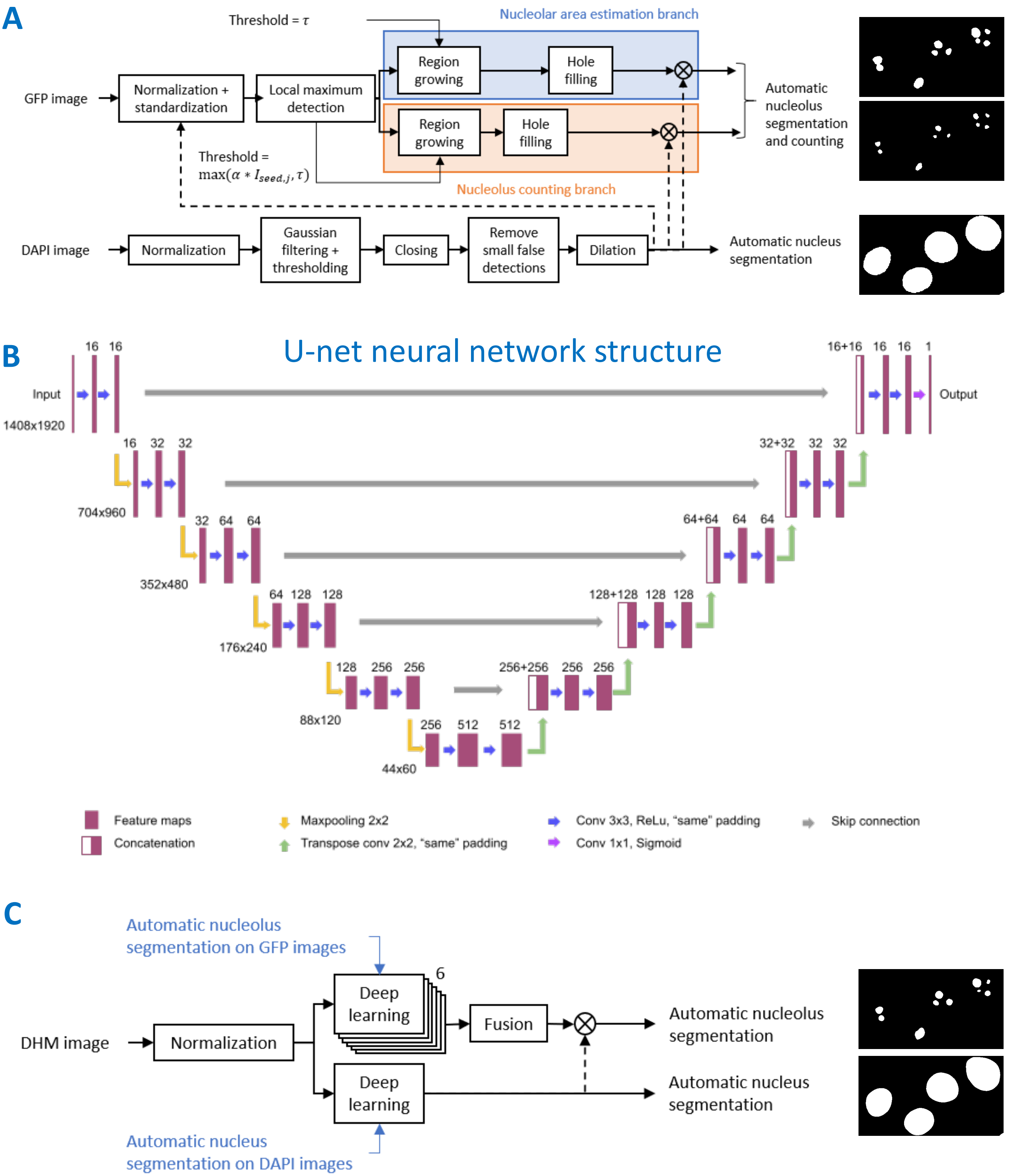

Appendix Figure S1. Image processing pipeline

A Segmentation pipeline of fluorescence images. The dynamic range of the GFP and DAPI images was normalized to $[0, 1]$. GFP images were further standardized image-wise. Seeds were automatically positioned on the pixels with local maximum intensity in the GFP images. Starting from those seeds, region-growing algorithms were used with a constant threshold for nucleolus area estimation and an adaptive threshold for counting. $I_{seed,j}$ is the pixel intensity of seed j, τ the constant, and α a scalar between 0 and 1. Unrealistic holes remaining in segmented nucleolar masks were filled in a postprocessing step. After normalization, the DAPI images were filtered with a Gaussian kernel and an adaptive threshold was applied. The threshold was adapted for every DAPI image on the basis of the background and foreground (i.e., nucleus) pixel intensity distributions. Further postprocessing of the segmented nuclear masks included a morphological closing operation, removal of false detections, and a final morphological dilation operation. The symbol $\otimes$ denotes pixel-wise multiplication between binary masks to ensure that only nucleolar pixels contained within the nuclear area were considered to be *bona fide* nucleoli. The binary mask output of fluorescence image segmentation is illustrated (insets).

B Architecture of the 2D U-net convolutional neural network. Convolution operators are filters that extract image patterns useful for segmentation. The analysis path (left part) allows capturing context, whereas the synthesis path (right path) and skip connections allow retrieving high output resolution.

C Segmentation pipeline of DHM images. Two neural networks were trained, one with binary masks of nucleoli obtained by segmentation of the GFP images, the other with binary masks of nuclei obtained by segmentation of the DAPI channel. The binary mask output of DHM image segmentation is illustrated (insets).

Appendix Figure S2

A

Number of nucleoli per cell	Number of cells	Measured nucleolar area (µm²)	Predicted nucleolar area (µm²)
1	238	22.62 ± 9.27	22.62
2	473	22.56 ± 7.48	≤ 28.49
3	331	22.72 ± 9.03	≤ 32.62
4	129	23.95 ± 13.10	≤ 35.91
5	25	31.21 ± 12.72	≤ 38.68
6	6	45.78 ± 9.67	≤ 41.10
7	4	48.93 ± 31.29	≤ 43.27

B

Counted on GFP images:

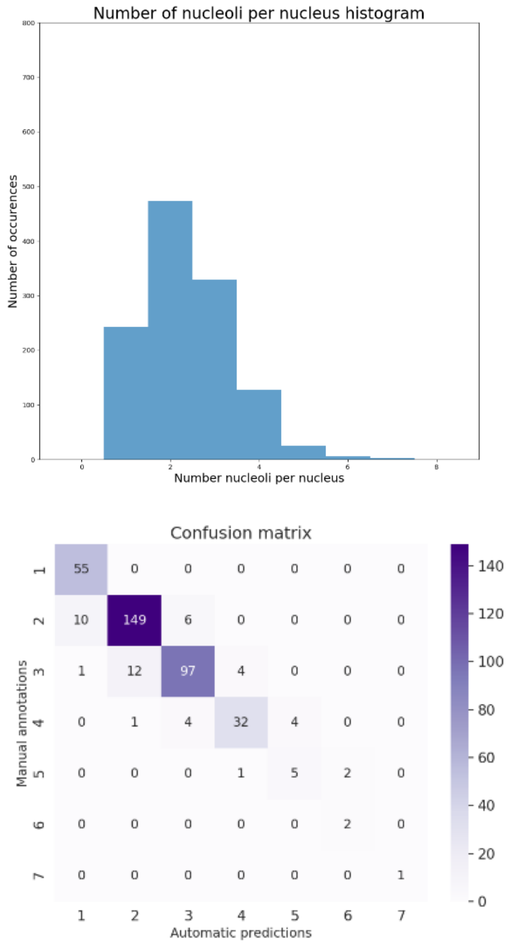

Counted on DHM images:

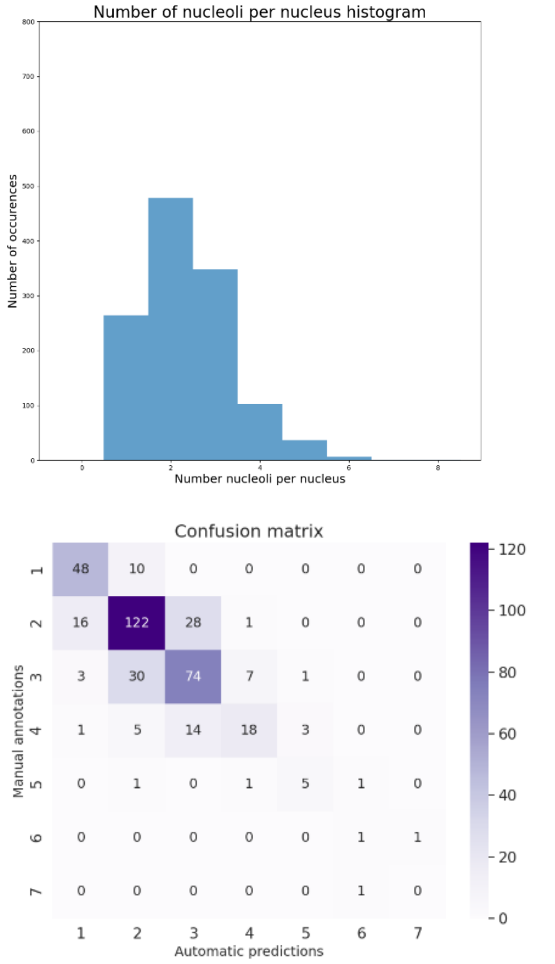

C

Number of nucleoli per cell	Fluorescence / Thresholding			DHM phase / Neural network		
	Sensitivity	Specificity	Precision	Sensitivity	Specificity	Precision
$n = 1$	1	0.97	0.83	0.83	0.94	0.71
$n = 2$	0.90	0.94	0.92	0.73	0.80	0.73
$n = 3$	0.85	0.96	0.91	0.64	0.85	0.64
$n = 4$	0.78	0.99	0.86	0.44	0.98	0.67

Appendix Figure S2. Benchmarking of data analysis

A Conservation of the mean nucleolar area according to the number of nucleoli per cell. The predicted value is a theoretical upper limit, considering that the area occupied by a single spherical nucleolus is multiplied by $N^{1/3}$ when the nucleolus is partitioned into N identical spheres.

B Histogram representing the number of nucleoli per nucleus automatically counted on the fluorescence images (left) and DHM images (right). Confusion matrices assessing automatic counting of nucleoli on the fluorescence images. The matrices compare the manual and automatic counts of nucleoli on 25 images. Element (i,j) in the confusion matrix is the number of nuclei with i nucleoli for which the algorithm counted j nucleoli. The elements on the main matrix diagonal were correctly counted. The lower the total value of elements outside the main diagonal, the better the counting.

C Sensitivity (true positive rate), specificity (true negative rate), and precision (proportion of properly classified cells) of the cell classification based on their number of nucleoli. Sensitivity provides the probability of counting n nucleoli amongst cells truly displaying n nucleoli. Specificity provides the probability of not counting n nucleoli amongst cells displaying n nucleoli. Precision provides the proportion of cells correctly classified. The closer the sensitivity, specificity, and precision are to 1, the better the approach.

Appendix Figure S3

A Segmentation by thresholding on GFP images:

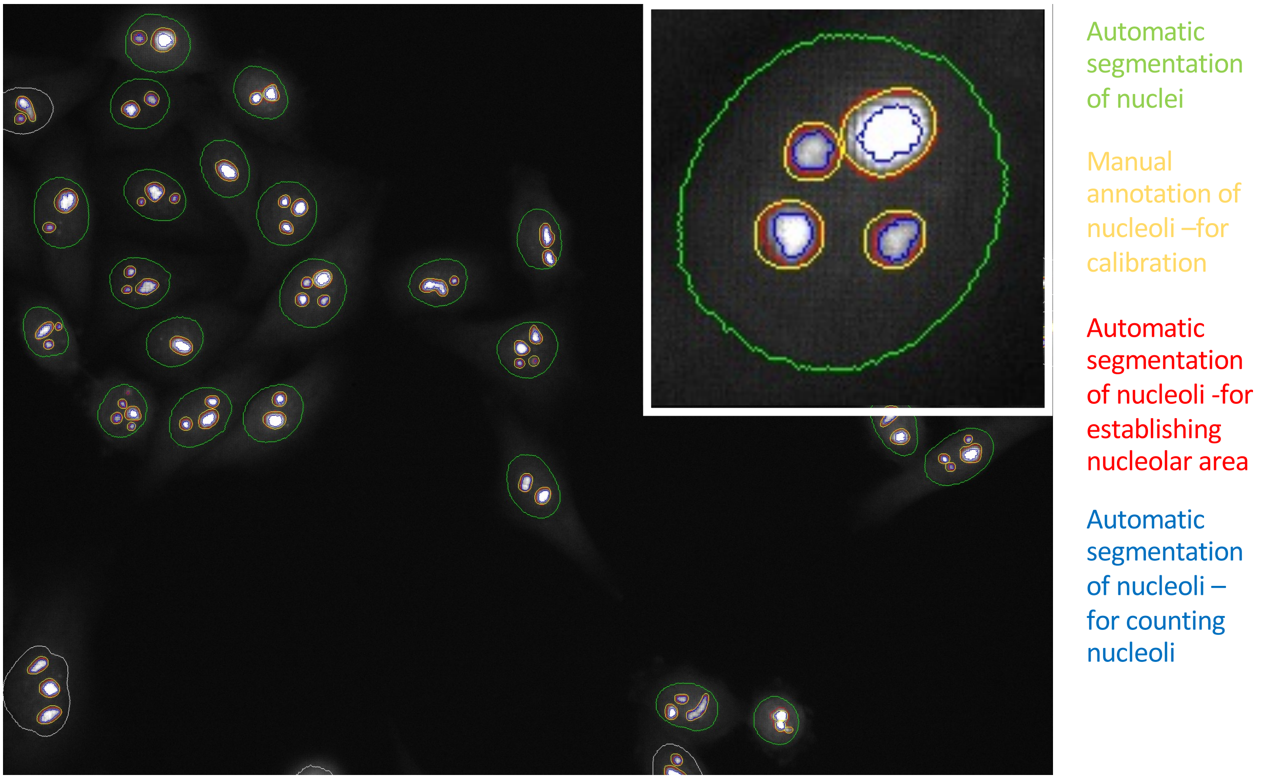

B Segmentation by deep learning on DHM images:

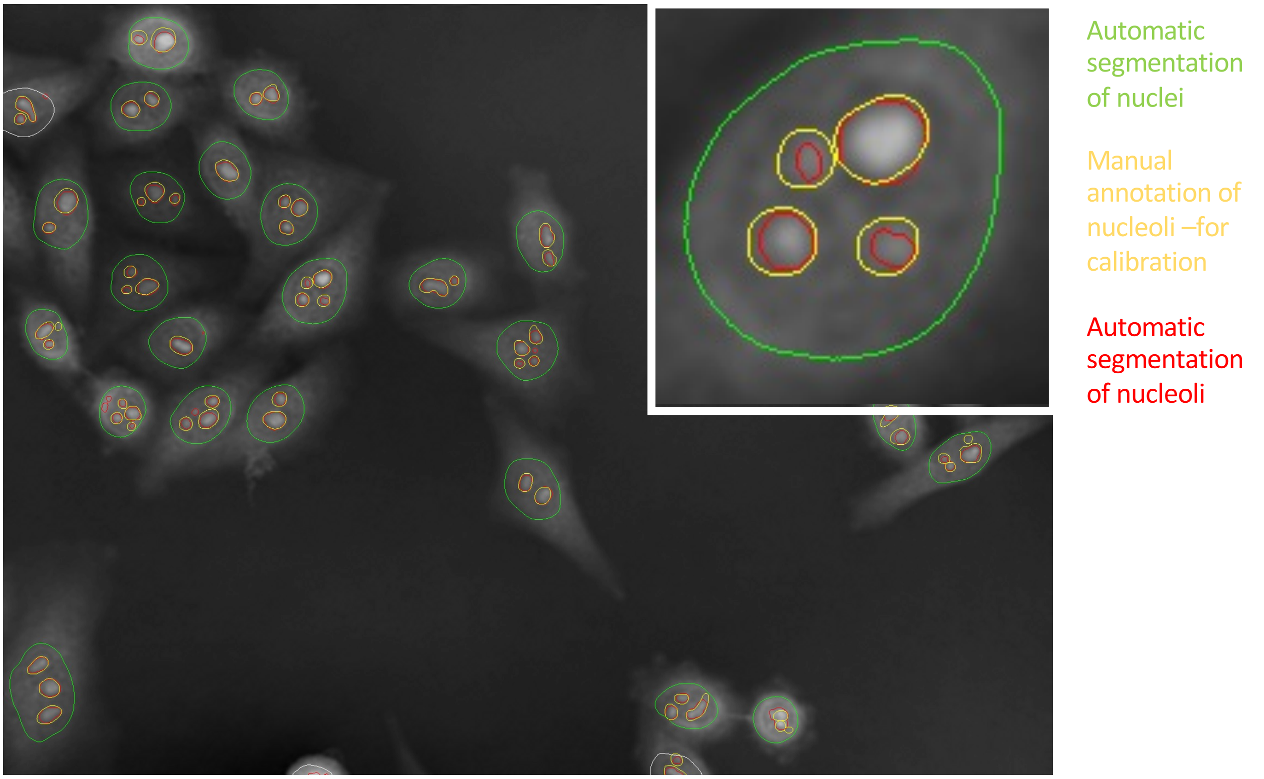

Appendix Figure S3. Comparison of segmentation of the GFP signal by thresholding and of the DHM signal by deep learning

A Segmentation of the GFP signal by thresholding. Red, automatic segmentation of nucleoli on GFP images by ‘region growing’, for computing the nucleolar area; blue: automatic segmentation of nucleoli by ‘region growing’, for counting; yellow: manual annotation for validation of the nucleolus counting algorithm; green: automatic segmentation of the nuclei on GFP images by thresholding; gray, eliminated cells (image edges, clustered cells, etc.).

B Segmentation of the DHM signal by deep learning. Red, automatic segmentation of nucleoli on DHM images by deep learning (for counting, area computation, and nucleolar optical thickness computation); green, automatic segmentation of the nuclei on DHM images by deep learning; yellow, same as in panel A.
