# Supplementary figures and images for: Detecting material state changes in the nucleolus by label-free digital holographic microscopy (part 1 of 2)
Source: EMBO Rep. 2024 Apr 23;25(6):2786–811. doi: 10.1038/s44319-024-00134-5 (PMC11169520; doi:10.1038/s44319-024-00134-5)

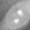

Supplement: Supplementary file 5 — Source data Fig. 2 [file 44319_2024_134_MOESM5_ESM.zip › Zorbas et al 2024_Source data_FIG 2/FIG2B/Fig2_B_Phase_inset.tif]

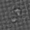

Supplement: Supplementary file 5 — Source data Fig. 2 [file 44319_2024_134_MOESM5_ESM.zip › Zorbas et al 2024_Source data_FIG 2/FIG2B/Fig2_B_Holo_inset.tif]

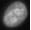

Supplement: Supplementary file 5 — Source data Fig. 2 [file 44319_2024_134_MOESM5_ESM.zip › Zorbas et al 2024_Source data_FIG 2/FIG2B/Fig2_B_DAPI_inset.tif]

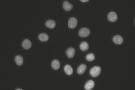

Supplement: Supplementary file 5 — Source data Fig. 2 [file 44319_2024_134_MOESM5_ESM.zip › Zorbas et al 2024_Source data_FIG 2/FIG2B/Fig2_B_DAPI.tif]

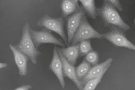

Supplement: Supplementary file 5 — Source data Fig. 2 [file 44319_2024_134_MOESM5_ESM.zip › Zorbas et al 2024_Source data_FIG 2/FIG2B/Fig2_B_Phase.tif]

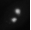

Supplement: Supplementary file 5 — Source data Fig. 2 [file 44319_2024_134_MOESM5_ESM.zip › Zorbas et al 2024_Source data_FIG 2/FIG2B/Fig2_B_GFP_inset.tif]

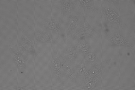

Supplement: Supplementary file 5 — Source data Fig. 2 [file 44319_2024_134_MOESM5_ESM.zip › Zorbas et al 2024_Source data_FIG 2/FIG2B/Fig2_B_Holo.tif]

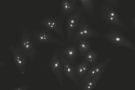

Supplement: Supplementary file 5 — Source data Fig. 2 [file 44319_2024_134_MOESM5_ESM.zip › Zorbas et al 2024_Source data_FIG 2/FIG2B/Fig2_B_GFP.tif]

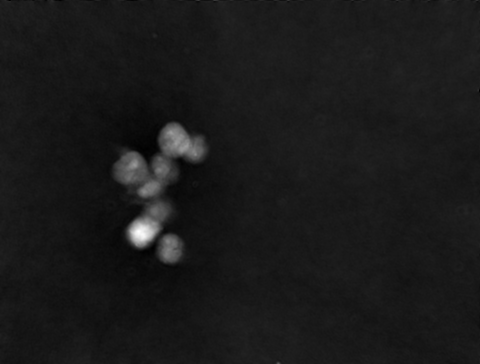

Supplement: Supplementary file 5 — Source data Fig. 2 [file 44319_2024_134_MOESM5_ESM.zip › Zorbas et al 2024_Source data_FIG 2/FIG2E/LCL_IF_anti_PES1_Phase.tif]

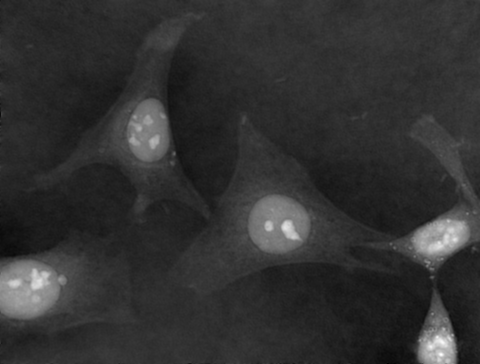

Supplement: Supplementary file 5 — Source data Fig. 2 [file 44319_2024_134_MOESM5_ESM.zip › Zorbas et al 2024_Source data_FIG 2/FIG2E/HeLa_IF_anti_PES1_Phase.tif]

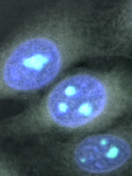

Supplement: Supplementary file 5 — Source data Fig. 2 [file 44319_2024_134_MOESM5_ESM.zip › Zorbas et al 2024_Source data_FIG 2/FIG2D/HeLa_FBLGFP_Merged.tif]

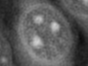

Supplement: Supplementary file 5 — Source data Fig. 2 [file 44319_2024_134_MOESM5_ESM.zip › Zorbas et al 2024_Source data_FIG 2/FIG2C/HeLa_FBL-GFP_phase_inset.png]

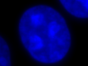

Supplement: Supplementary file 5 — Source data Fig. 2 [file 44319_2024_134_MOESM5_ESM.zip › Zorbas et al 2024_Source data_FIG 2/FIG2C/HeLa_FBL-GFP_DAPI_inset.tif]

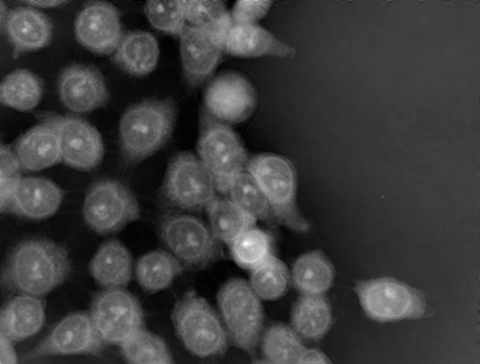

Supplement: Supplementary file 5 — Source data Fig. 2 [file 44319_2024_134_MOESM5_ESM.zip › Zorbas et al 2024_Source data_FIG 2/FIG2C/HCT116_FBL-GFP_phase.tif]

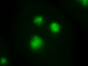

Supplement: Supplementary file 5 — Source data Fig. 2 [file 44319_2024_134_MOESM5_ESM.zip › Zorbas et al 2024_Source data_FIG 2/FIG2C/HeLa_FBL-GFP_GFP_inset.tif]

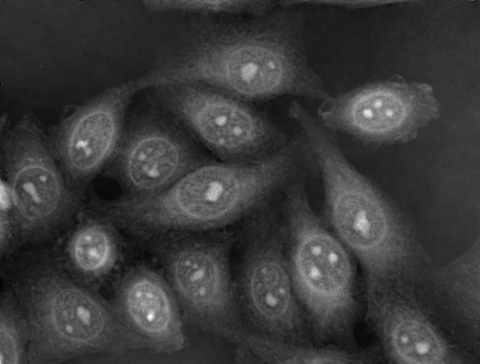

Supplement: Supplementary file 5 — Source data Fig. 2 [file 44319_2024_134_MOESM5_ESM.zip › Zorbas et al 2024_Source data_FIG 2/FIG2C/HeLa_FBL-GFP_phase.tif]

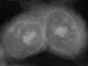

Supplement: Supplementary file 5 — Source data Fig. 2 [file 44319_2024_134_MOESM5_ESM.zip › Zorbas et al 2024_Source data_FIG 2/FIG2C/HCT116_FBL-GFP_phase_inset.tif]

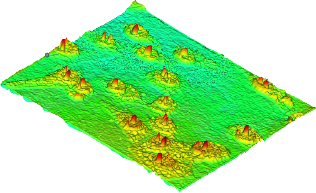

Supplement: Supplementary file 5 — Source data Fig. 2 [file 44319_2024_134_MOESM5_ESM.zip › Zorbas et al 2024_Source data_FIG 2/FIG2A/FIG2A_heatmap.tif]

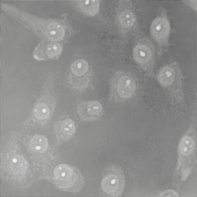

Supplement: Supplementary file 5 — Source data Fig. 2 [file 44319_2024_134_MOESM5_ESM.zip › Zorbas et al 2024_Source data_FIG 2/FIG2A/FIG2A.tif]

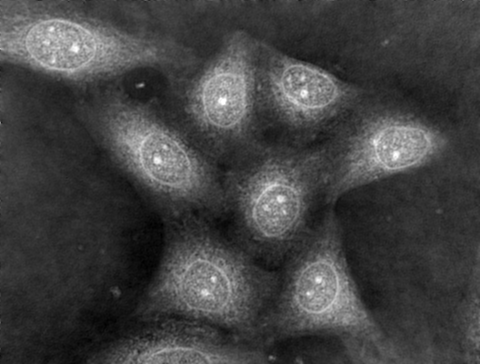

Supplement: Supplementary file 6 — Source data Fig. 3 [file 44319_2024_134_MOESM6_ESM.zip › Zorbas et al 2024_Source data_FIG 3/FIG3A/CX5461 Phase.tif]

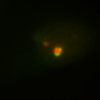

Supplement: Supplementary file 6 — Source data Fig. 3 [file 44319_2024_134_MOESM6_ESM.zip › Zorbas et al 2024_Source data_FIG 3/FIG3A/CX5461 IF Inset.tif]

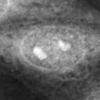

Supplement: Supplementary file 6 — Source data Fig. 3 [file 44319_2024_134_MOESM6_ESM.zip › Zorbas et al 2024_Source data_FIG 3/FIG3A/DMSO Phase Inset.tif]

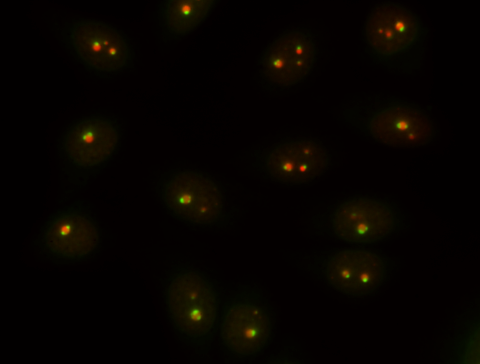

Supplement: Supplementary file 6 — Source data Fig. 3 [file 44319_2024_134_MOESM6_ESM.zip › Zorbas et al 2024_Source data_FIG 3/FIG3A/ActD IF.tif]

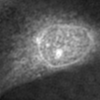

Supplement: Supplementary file 6 — Source data Fig. 3 [file 44319_2024_134_MOESM6_ESM.zip › Zorbas et al 2024_Source data_FIG 3/FIG3A/CX5461 Phase Inset.tif]

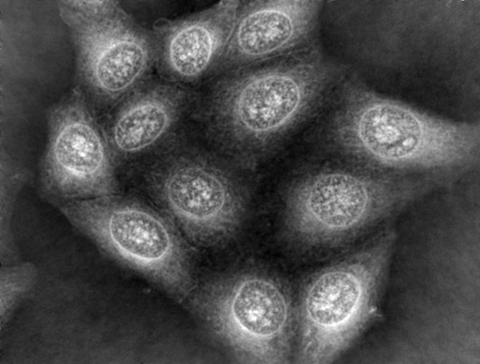

Supplement: Supplementary file 6 — Source data Fig. 3 [file 44319_2024_134_MOESM6_ESM.zip › Zorbas et al 2024_Source data_FIG 3/FIG3A/DRB Phase.tif]

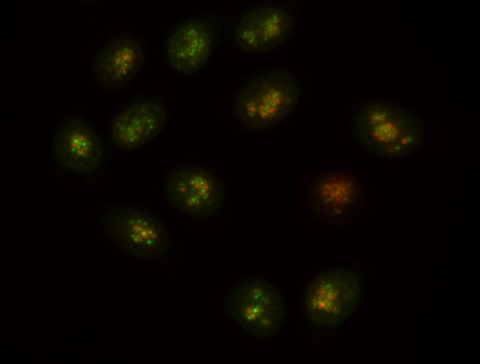

Supplement: Supplementary file 6 — Source data Fig. 3 [file 44319_2024_134_MOESM6_ESM.zip › Zorbas et al 2024_Source data_FIG 3/FIG3A/DRB IF.tif]

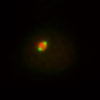

Supplement: Supplementary file 6 — Source data Fig. 3 [file 44319_2024_134_MOESM6_ESM.zip › Zorbas et al 2024_Source data_FIG 3/FIG3A/ActD IF Inset.tif]

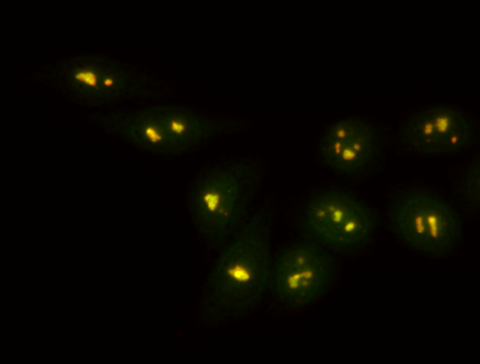

Supplement: Supplementary file 6 — Source data Fig. 3 [file 44319_2024_134_MOESM6_ESM.zip › Zorbas et al 2024_Source data_FIG 3/FIG3A/DMSO IF.tif]

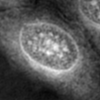

Supplement: Supplementary file 6 — Source data Fig. 3 [file 44319_2024_134_MOESM6_ESM.zip › Zorbas et al 2024_Source data_FIG 3/FIG3A/DRB Phase Inset.tif]

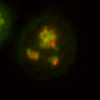

Supplement: Supplementary file 6 — Source data Fig. 3 [file 44319_2024_134_MOESM6_ESM.zip › Zorbas et al 2024_Source data_FIG 3/FIG3A/Roscovitine IF Inset.tif]

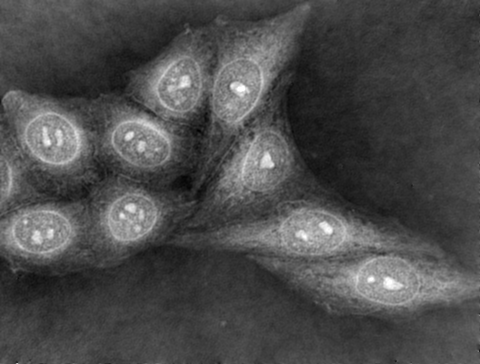

Supplement: Supplementary file 6 — Source data Fig. 3 [file 44319_2024_134_MOESM6_ESM.zip › Zorbas et al 2024_Source data_FIG 3/FIG3A/DMSO Phase.tif]

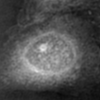

Supplement: Supplementary file 6 — Source data Fig. 3 [file 44319_2024_134_MOESM6_ESM.zip › Zorbas et al 2024_Source data_FIG 3/FIG3A/ActD Phase Inset.tif]

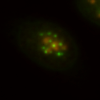

Supplement: Supplementary file 6 — Source data Fig. 3 [file 44319_2024_134_MOESM6_ESM.zip › Zorbas et al 2024_Source data_FIG 3/FIG3A/DRB IF Inset.tif]

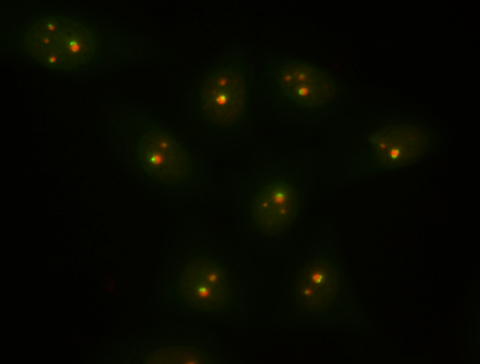

Supplement: Supplementary file 6 — Source data Fig. 3 [file 44319_2024_134_MOESM6_ESM.zip › Zorbas et al 2024_Source data_FIG 3/FIG3A/CX5461 IF.tif]

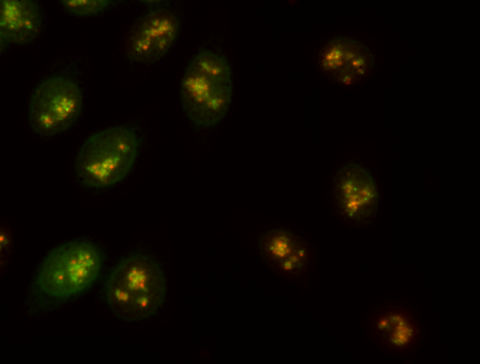

Supplement: Supplementary file 6 — Source data Fig. 3 [file 44319_2024_134_MOESM6_ESM.zip › Zorbas et al 2024_Source data_FIG 3/FIG3A/Roscovitine IF.tif]

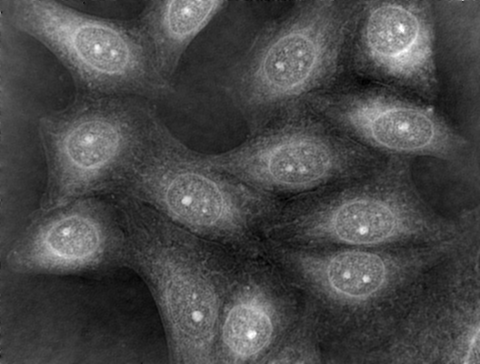

Supplement: Supplementary file 6 — Source data Fig. 3 [file 44319_2024_134_MOESM6_ESM.zip › Zorbas et al 2024_Source data_FIG 3/FIG3A/ActD Phase.tif]

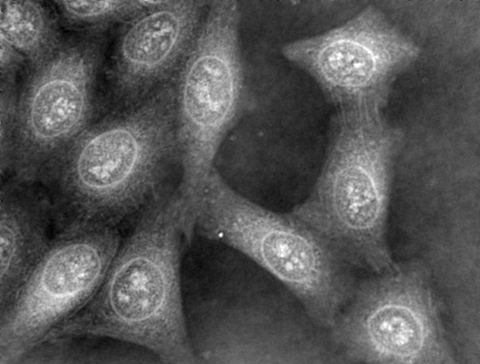

Supplement: Supplementary file 6 — Source data Fig. 3 [file 44319_2024_134_MOESM6_ESM.zip › Zorbas et al 2024_Source data_FIG 3/FIG3A/Roscovitine Phase.tif]

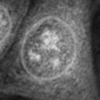

Supplement: Supplementary file 6 — Source data Fig. 3 [file 44319_2024_134_MOESM6_ESM.zip › Zorbas et al 2024_Source data_FIG 3/FIG3A/Roscovitine Phase Inset.tif]

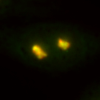

Supplement: Supplementary file 6 — Source data Fig. 3 [file 44319_2024_134_MOESM6_ESM.zip › Zorbas et al 2024_Source data_FIG 3/FIG3A/DMSO IF Inset.tif]

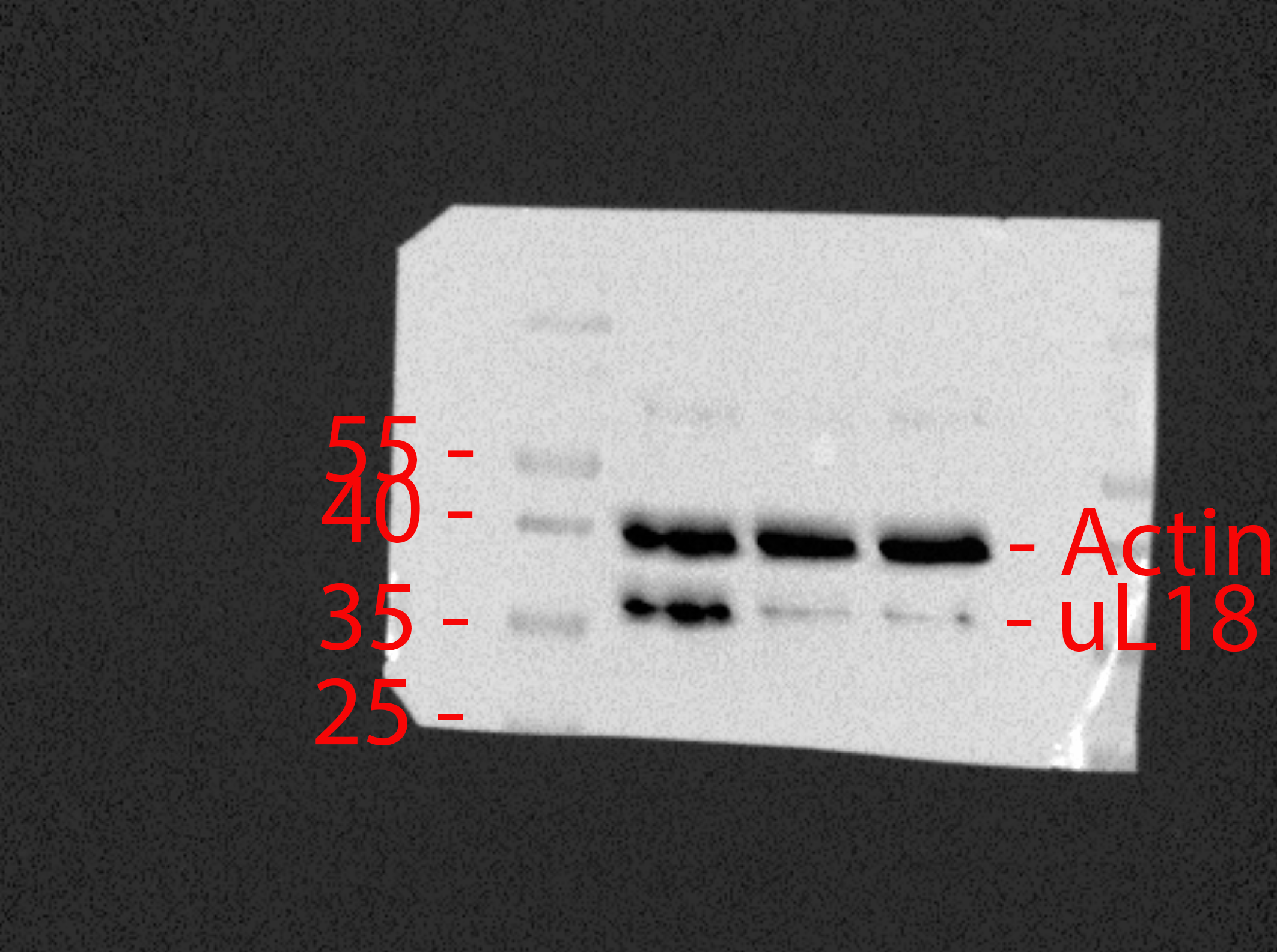

Supplement: Supplementary file 6 — Source data Fig. 3 [file 44319_2024_134_MOESM6_ESM.zip › Zorbas et al 2024_Source data_FIG 3/FIG3D/Actin_replicate_after_ul18_hybridization.tif]

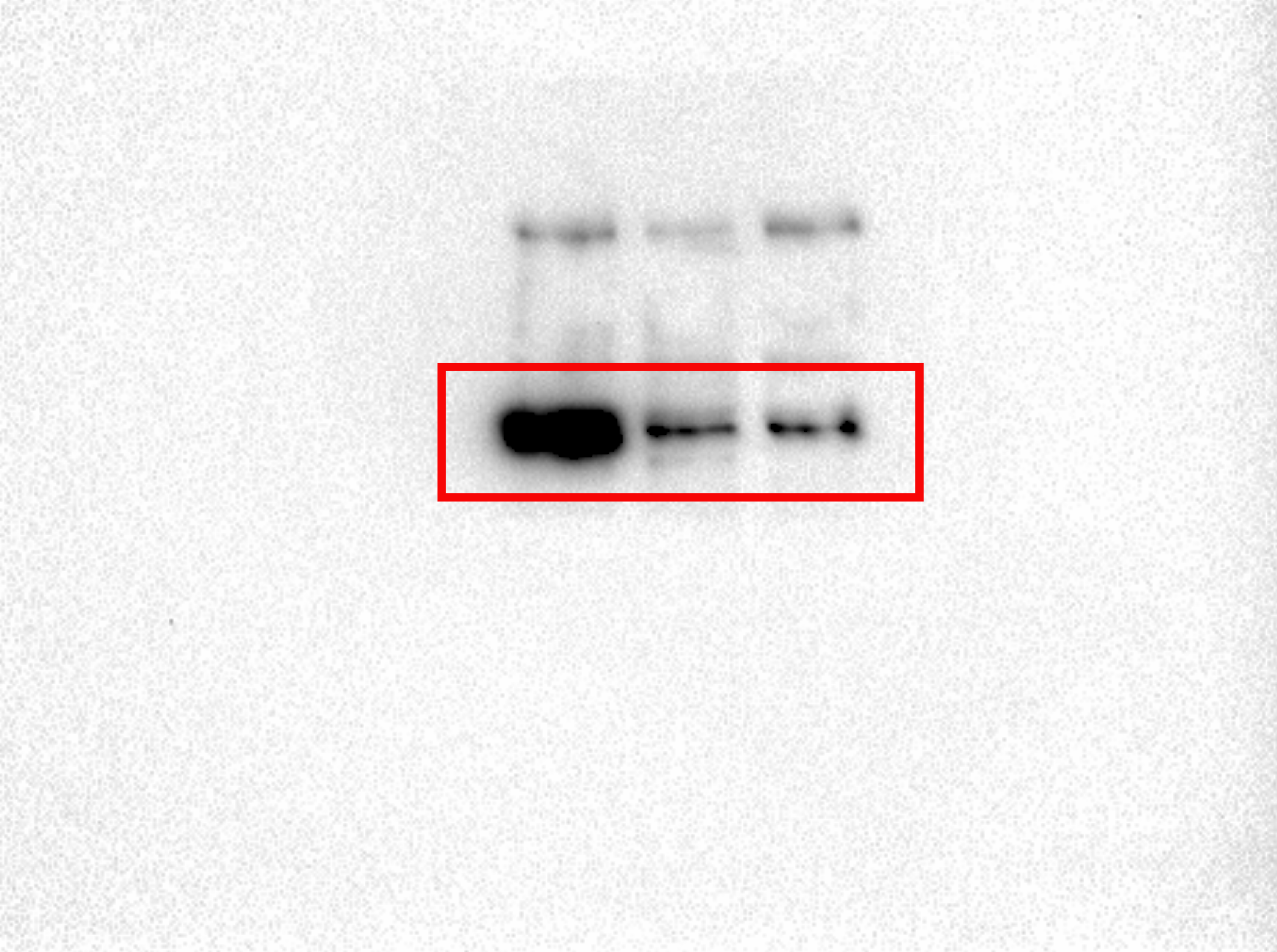

Supplement: Supplementary file 6 — Source data Fig. 3 [file 44319_2024_134_MOESM6_ESM.zip › Zorbas et al 2024_Source data_FIG 3/FIG3D/FIG3_D_uL18.tif]

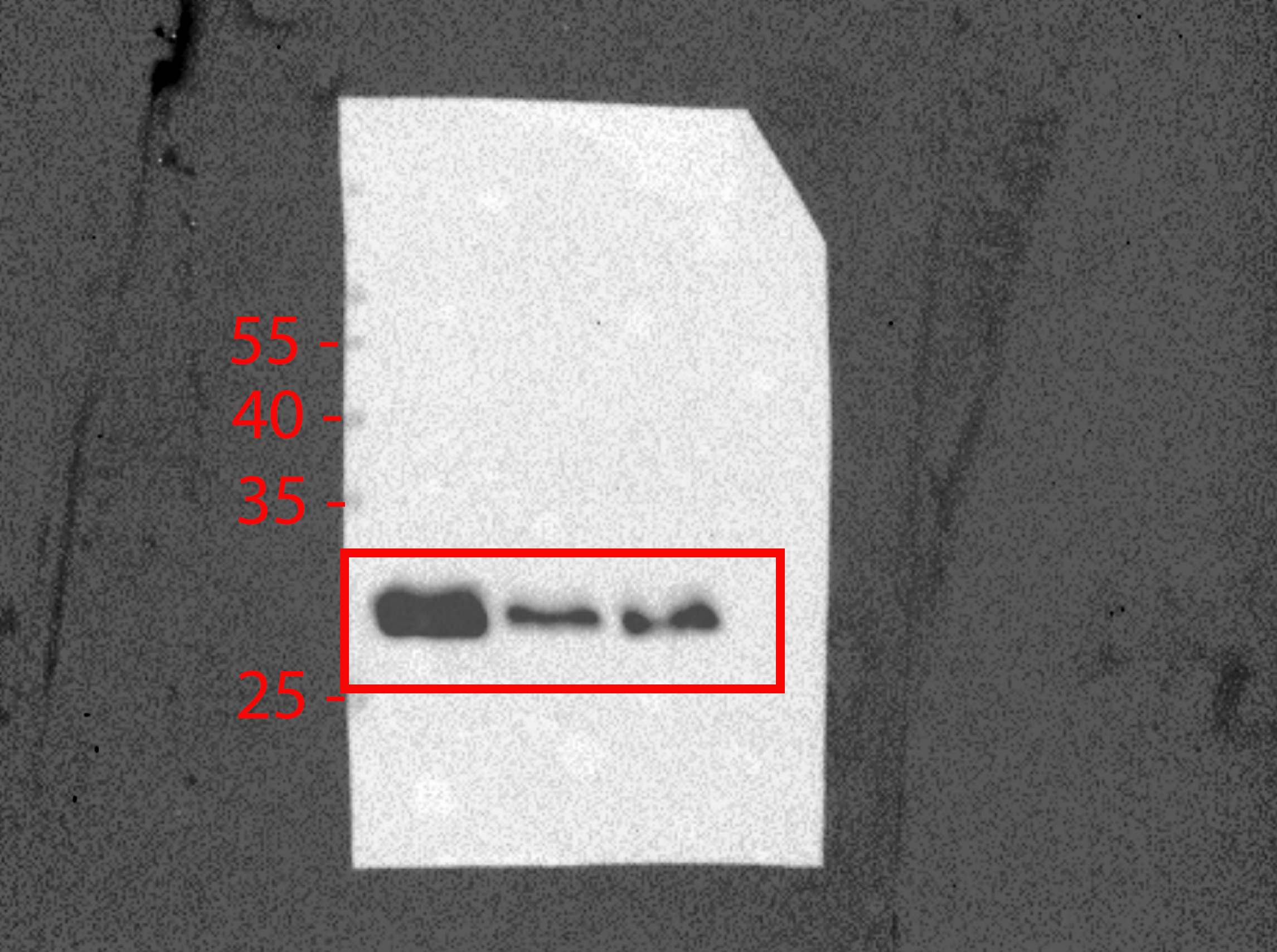

Supplement: Supplementary file 6 — Source data Fig. 3 [file 44319_2024_134_MOESM6_ESM.zip › Zorbas et al 2024_Source data_FIG 3/FIG3D/FIG3_D_uL5.tif]

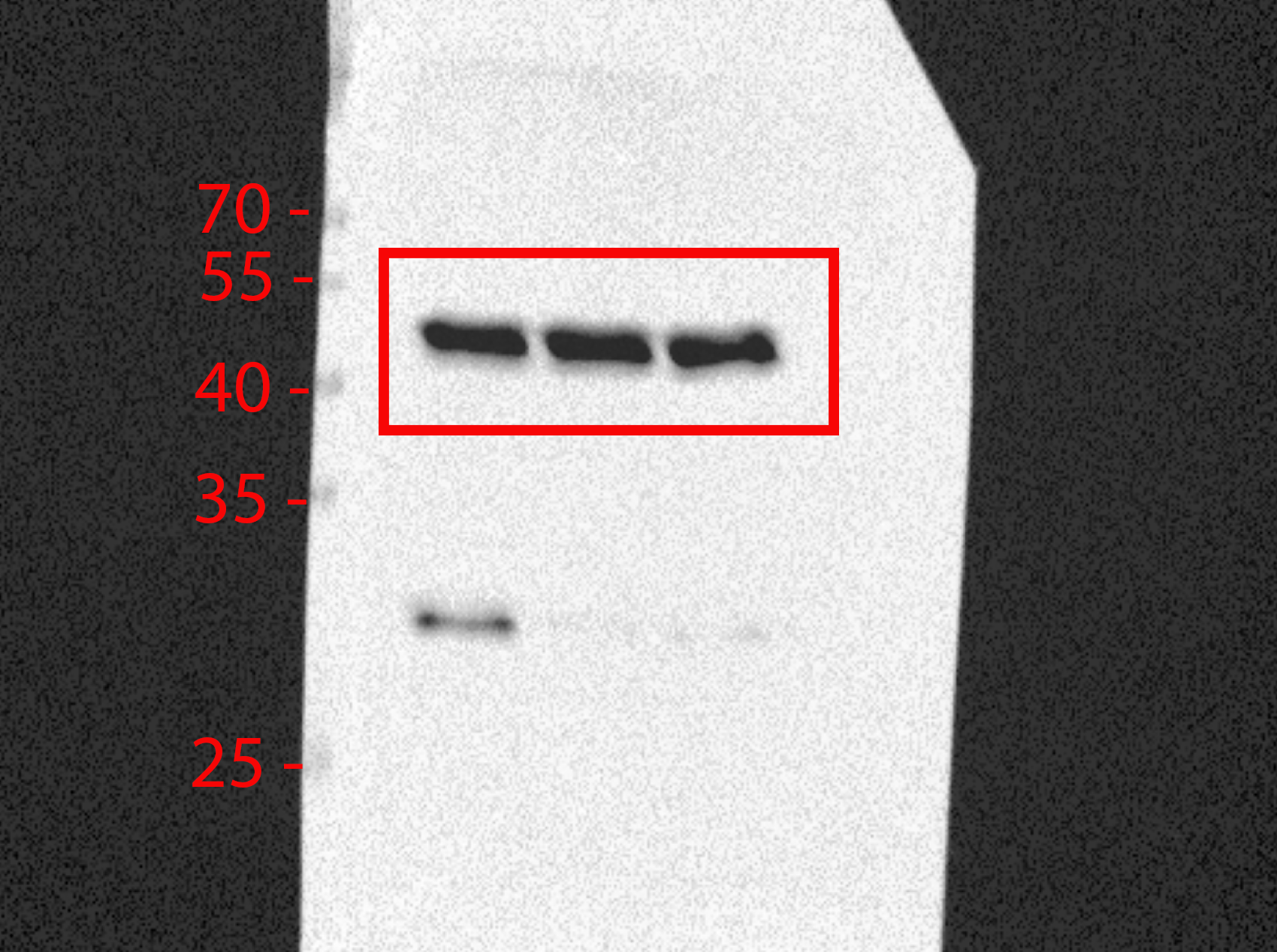

Supplement: Supplementary file 6 — Source data Fig. 3 [file 44319_2024_134_MOESM6_ESM.zip › Zorbas et al 2024_Source data_FIG 3/FIG3D/FIG3_D_Actin.tif]

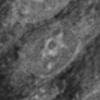

Supplement: Supplementary file 6 — Source data Fig. 3 [file 44319_2024_134_MOESM6_ESM.zip › Zorbas et al 2024_Source data_FIG 3/FIG3C/uL18_Phase_inset.tif]

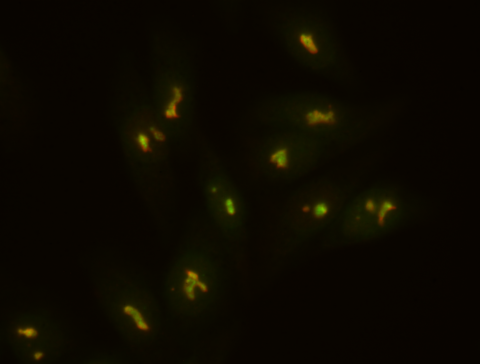

Supplement: Supplementary file 6 — Source data Fig. 3 [file 44319_2024_134_MOESM6_ESM.zip › Zorbas et al 2024_Source data_FIG 3/FIG3C/uL5_FBL-PES1.tif]

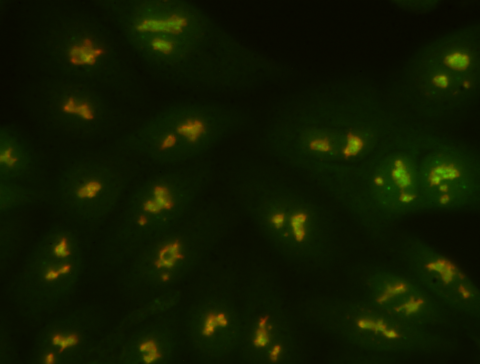

Supplement: Supplementary file 6 — Source data Fig. 3 [file 44319_2024_134_MOESM6_ESM.zip › Zorbas et al 2024_Source data_FIG 3/FIG3C/uL18_FBL-PES1.tif]

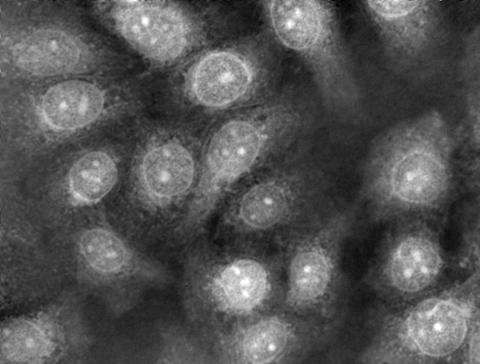

Supplement: Supplementary file 6 — Source data Fig. 3 [file 44319_2024_134_MOESM6_ESM.zip › Zorbas et al 2024_Source data_FIG 3/FIG3C/SCR_Phase.tif]

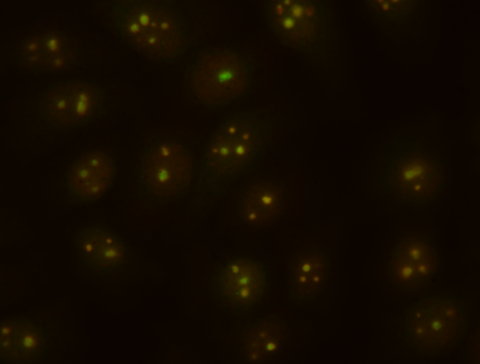

Supplement: Supplementary file 6 — Source data Fig. 3 [file 44319_2024_134_MOESM6_ESM.zip › Zorbas et al 2024_Source data_FIG 3/FIG3C/SCR_FBL-PES1.tif]

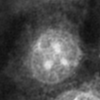

Supplement: Supplementary file 6 — Source data Fig. 3 [file 44319_2024_134_MOESM6_ESM.zip › Zorbas et al 2024_Source data_FIG 3/FIG3C/SCR_Phase_inset.tif]

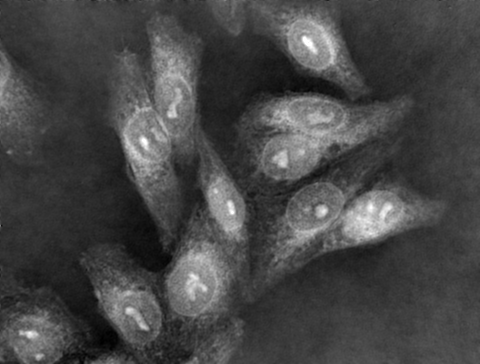

Supplement: Supplementary file 6 — Source data Fig. 3 [file 44319_2024_134_MOESM6_ESM.zip › Zorbas et al 2024_Source data_FIG 3/FIG3C/uL5_Phase.tif]

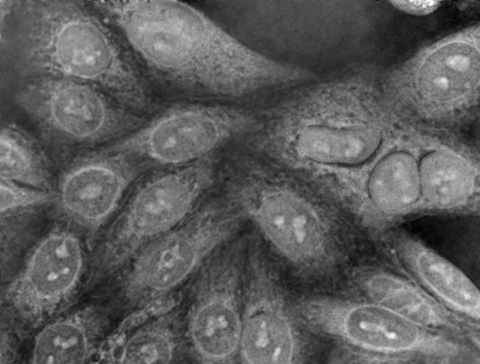

Supplement: Supplementary file 6 — Source data Fig. 3 [file 44319_2024_134_MOESM6_ESM.zip › Zorbas et al 2024_Source data_FIG 3/FIG3C/uL18_Phase.tif]

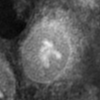

Supplement: Supplementary file 6 — Source data Fig. 3 [file 44319_2024_134_MOESM6_ESM.zip › Zorbas et al 2024_Source data_FIG 3/FIG3C/uL5_Phase_inset.tif]

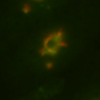

Supplement: Supplementary file 6 — Source data Fig. 3 [file 44319_2024_134_MOESM6_ESM.zip › Zorbas et al 2024_Source data_FIG 3/FIG3C/uL18_FBL-PES1_inset.tif]

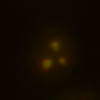

Supplement: Supplementary file 6 — Source data Fig. 3 [file 44319_2024_134_MOESM6_ESM.zip › Zorbas et al 2024_Source data_FIG 3/FIG3C/SCR_FBL-PES1_inset.tif]

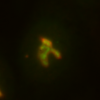

Supplement: Supplementary file 6 — Source data Fig. 3 [file 44319_2024_134_MOESM6_ESM.zip › Zorbas et al 2024_Source data_FIG 3/FIG3C/uL5_FBL-PES1_inset.tif]

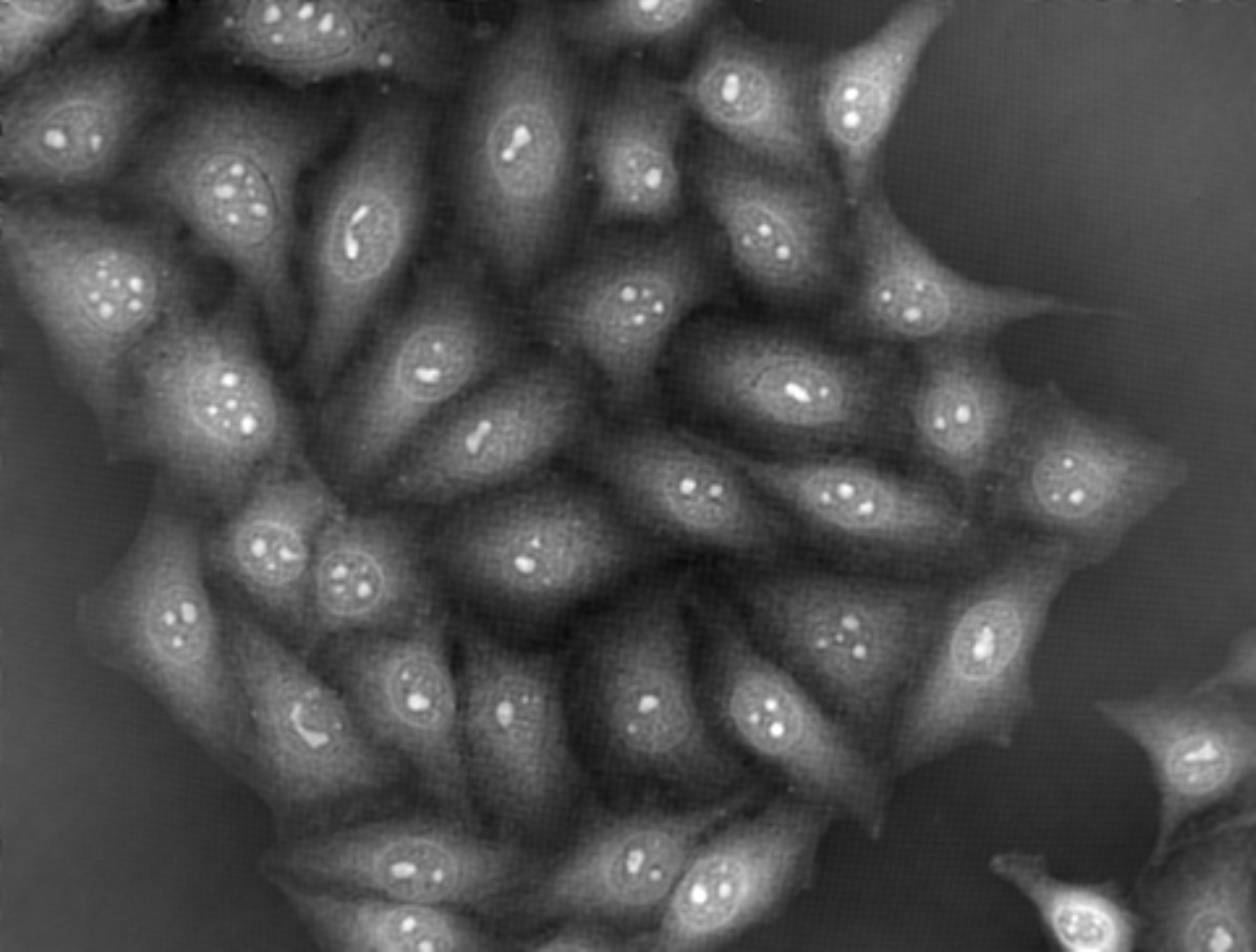

Supplement: Supplementary file 7 — Source data Fig. 4 [file 44319_2024_134_MOESM7_ESM.zip › Zorbas et al 2024_Source data_FIG 4/FIG4A/DHM Phase.tif]

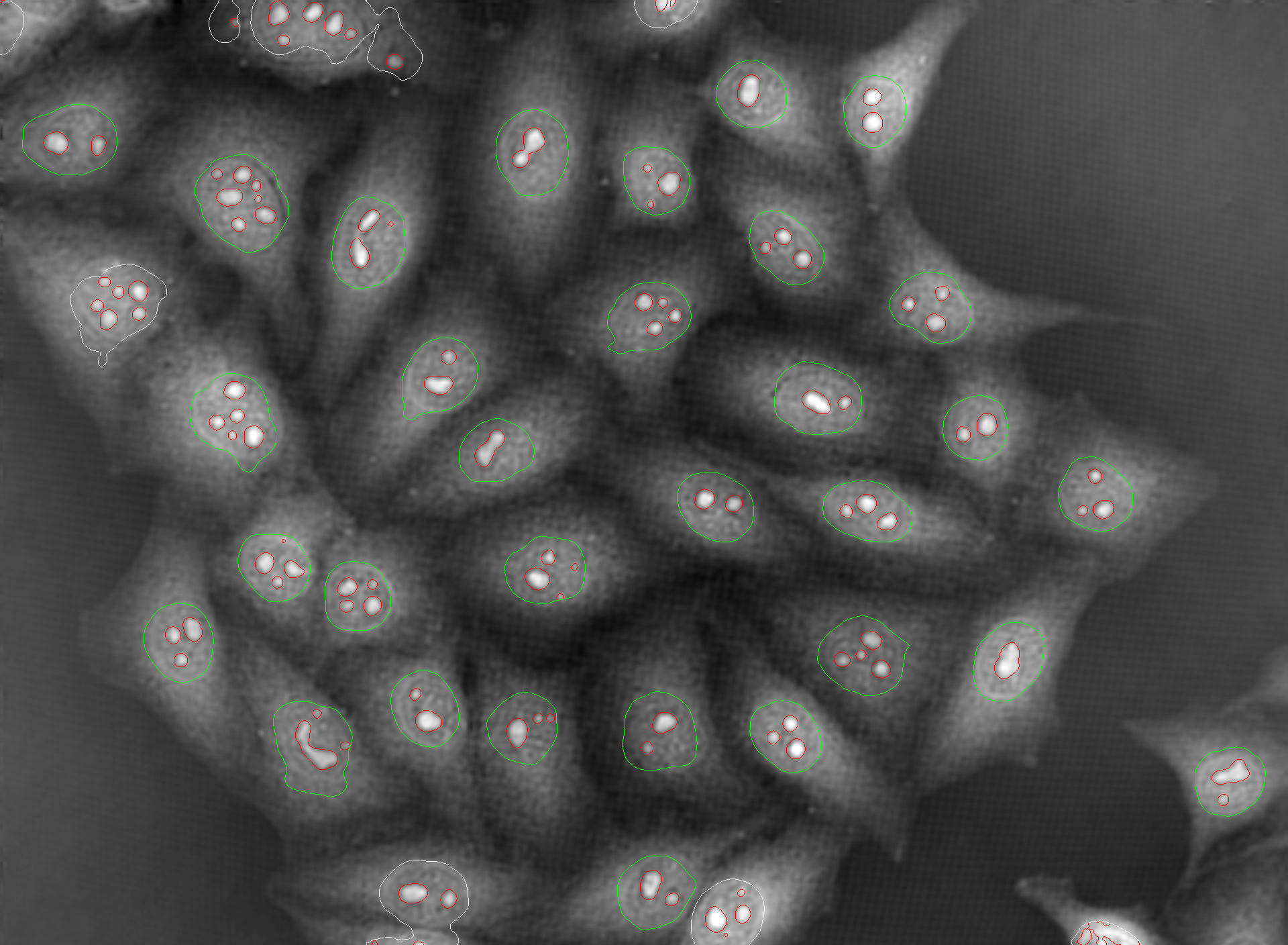

Supplement: Supplementary file 7 — Source data Fig. 4 [file 44319_2024_134_MOESM7_ESM.zip › Zorbas et al 2024_Source data_FIG 4/FIG4A/Deep learning.tif]

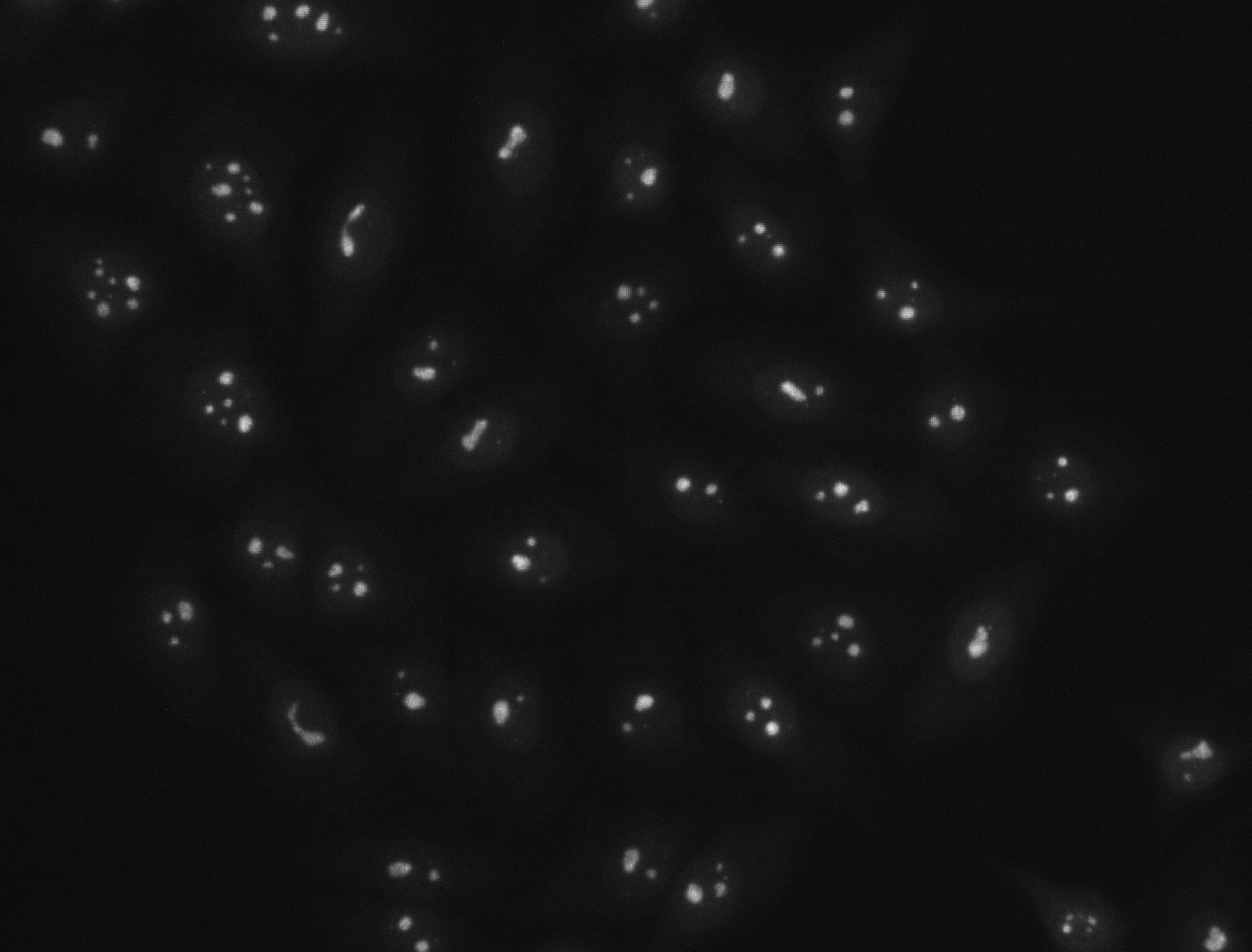

Supplement: Supplementary file 7 — Source data Fig. 4 [file 44319_2024_134_MOESM7_ESM.zip › Zorbas et al 2024_Source data_FIG 4/FIG4A/GFP.tif]

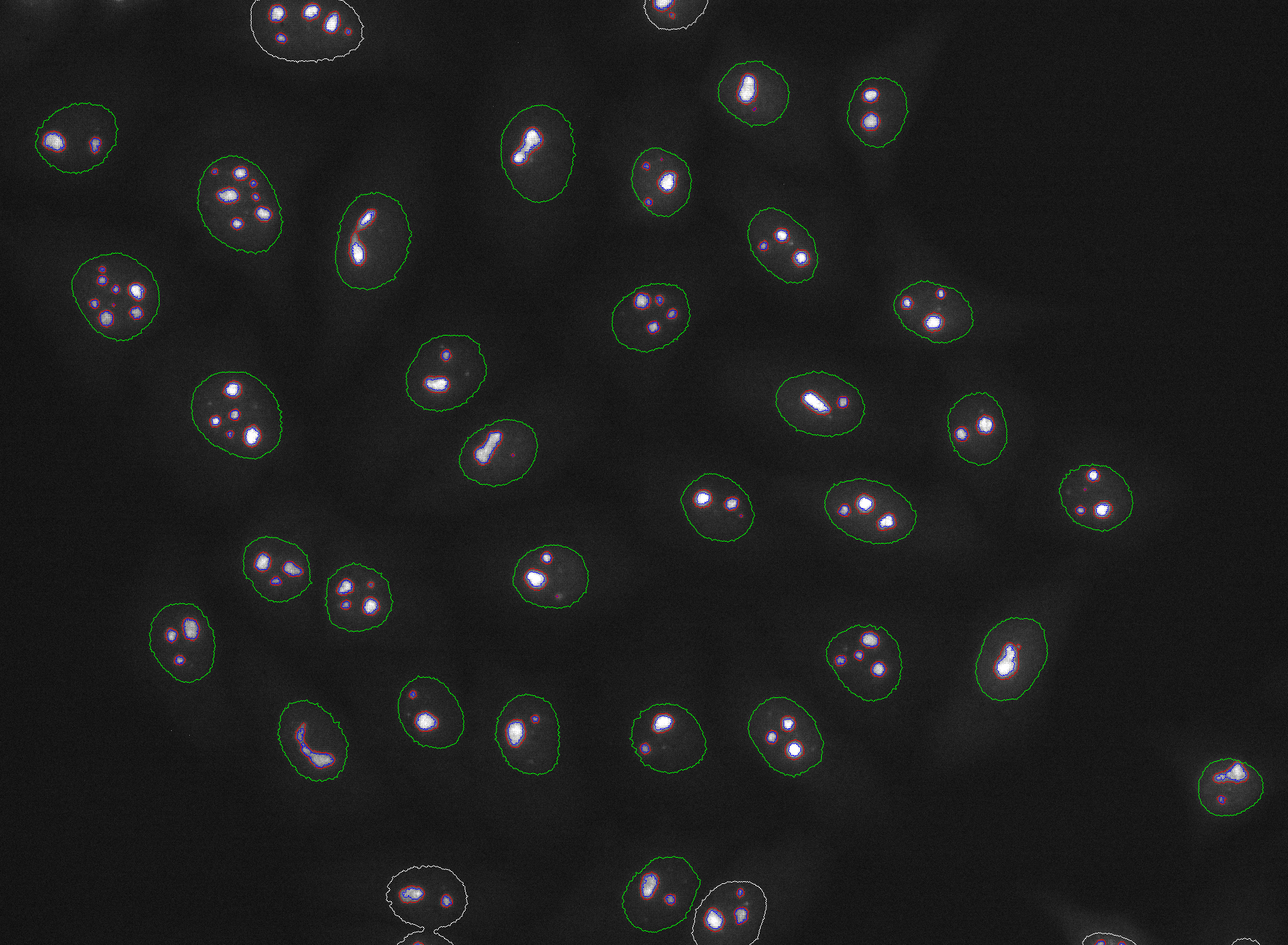

Supplement: Supplementary file 7 — Source data Fig. 4 [file 44319_2024_134_MOESM7_ESM.zip › Zorbas et al 2024_Source data_FIG 4/FIG4A/Thresholding.tif]

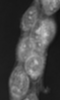

Supplement: Supplementary file 8 — Source data Fig. 5 [file 44319_2024_134_MOESM8_ESM.zip › Zorbas et al 2024_Source data_FIG 5/FIG5E/Medium BL.tif]

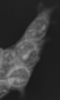

Supplement: Supplementary file 8 — Source data Fig. 5 [file 44319_2024_134_MOESM8_ESM.zip › Zorbas et al 2024_Source data_FIG 5/FIG5E/No BL.tif]

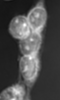

Supplement: Supplementary file 8 — Source data Fig. 5 [file 44319_2024_134_MOESM8_ESM.zip › Zorbas et al 2024_Source data_FIG 5/FIG5E/High BL.tif]

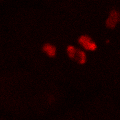

Supplement: Supplementary file 8 — Source data Fig. 5 [file 44319_2024_134_MOESM8_ESM.zip › Zorbas et al 2024_Source data_FIG 5/FIG5C/Medium BL t=60.tif]

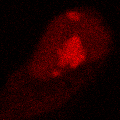

Supplement: Supplementary file 8 — Source data Fig. 5 [file 44319_2024_134_MOESM8_ESM.zip › Zorbas et al 2024_Source data_FIG 5/FIG5C/No BL t=60.tif]

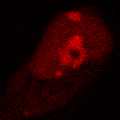

Supplement: Supplementary file 8 — Source data Fig. 5 [file 44319_2024_134_MOESM8_ESM.zip › Zorbas et al 2024_Source data_FIG 5/FIG5C/No BL bleach.tif]

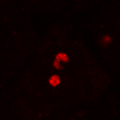

Supplement: Supplementary file 8 — Source data Fig. 5 [file 44319_2024_134_MOESM8_ESM.zip › Zorbas et al 2024_Source data_FIG 5/FIG5C/High BL bleach.tif]

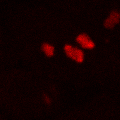

Supplement: Supplementary file 8 — Source data Fig. 5 [file 44319_2024_134_MOESM8_ESM.zip › Zorbas et al 2024_Source data_FIG 5/FIG5C/Medium BL t=0.tif]

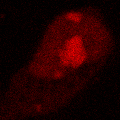

Supplement: Supplementary file 8 — Source data Fig. 5 [file 44319_2024_134_MOESM8_ESM.zip › Zorbas et al 2024_Source data_FIG 5/FIG5C/No BL t=0.tif]

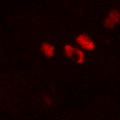

Supplement: Supplementary file 8 — Source data Fig. 5 [file 44319_2024_134_MOESM8_ESM.zip › Zorbas et al 2024_Source data_FIG 5/FIG5C/Medium BL bleach.tif]

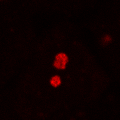

Supplement: Supplementary file 8 — Source data Fig. 5 [file 44319_2024_134_MOESM8_ESM.zip › Zorbas et al 2024_Source data_FIG 5/FIG5C/High BL t=0.tif]

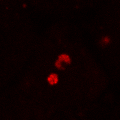

Supplement: Supplementary file 8 — Source data Fig. 5 [file 44319_2024_134_MOESM8_ESM.zip › Zorbas et al 2024_Source data_FIG 5/FIG5C/High BL t=60.tif]

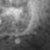

Supplement: Supplementary file 9 — Source data Fig. 6 [file 44319_2024_134_MOESM9_ESM.zip › Zorbas et al 2024_Source data_FIG 6/TDP43 Phase zoom.tif]

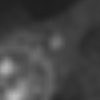

Supplement: Supplementary file 9 — Source data Fig. 6 [file 44319_2024_134_MOESM9_ESM.zip › Zorbas et al 2024_Source data_FIG 6/Ataxin3 image2 Phase zoom.tif]

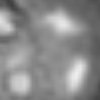

Supplement: Supplementary file 9 — Source data Fig. 6 [file 44319_2024_134_MOESM9_ESM.zip › Zorbas et al 2024_Source data_FIG 6/mHTT image1 Phase zoom.tif]

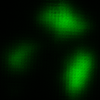

Supplement: Supplementary file 9 — Source data Fig. 6 [file 44319_2024_134_MOESM9_ESM.zip › Zorbas et al 2024_Source data_FIG 6/mHTT image1 GFP zoom.tif]

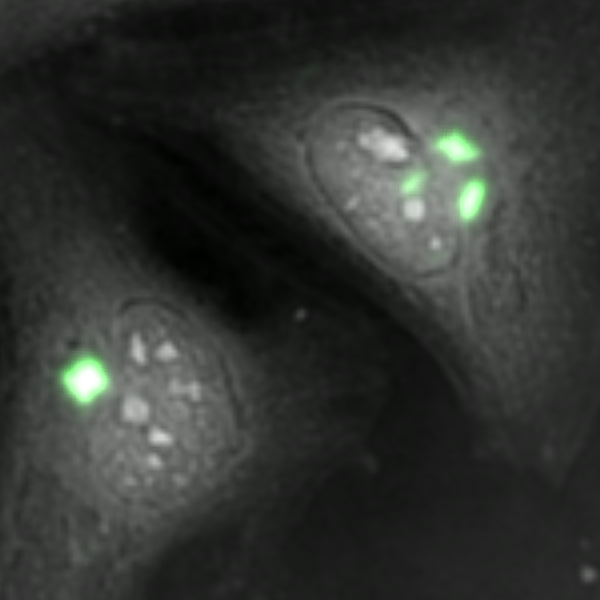

Supplement: Supplementary file 9 — Source data Fig. 6 [file 44319_2024_134_MOESM9_ESM.zip › Zorbas et al 2024_Source data_FIG 6/mHTT image1 overlay.tif]

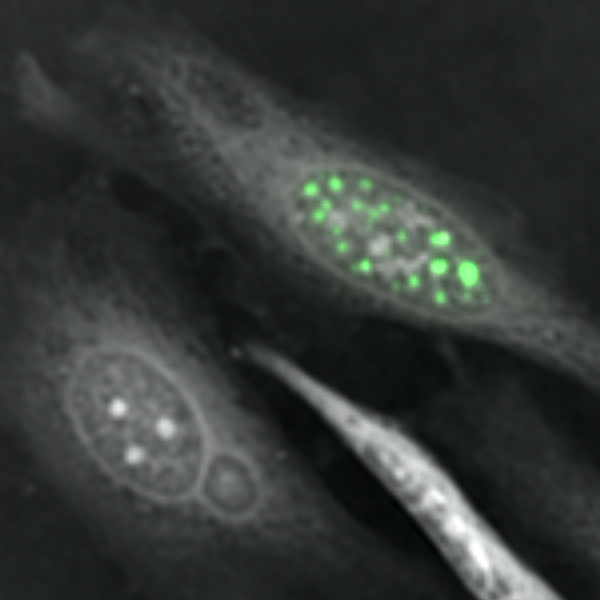

Supplement: Supplementary file 9 — Source data Fig. 6 [file 44319_2024_134_MOESM9_ESM.zip › Zorbas et al 2024_Source data_FIG 6/mHTT image2 overlay.tif]

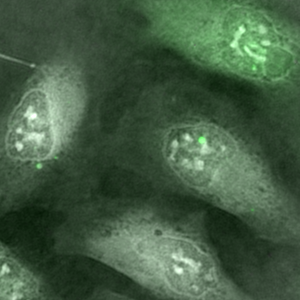

Supplement: Supplementary file 9 — Source data Fig. 6 [file 44319_2024_134_MOESM9_ESM.zip › Zorbas et al 2024_Source data_FIG 6/TDP43 overlay.tif]

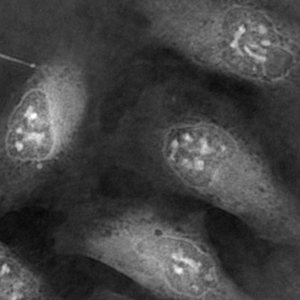

Supplement: Supplementary file 9 — Source data Fig. 6 [file 44319_2024_134_MOESM9_ESM.zip › Zorbas et al 2024_Source data_FIG 6/TDP43 Phase.tif]

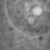

Supplement: Supplementary file 9 — Source data Fig. 6 [file 44319_2024_134_MOESM9_ESM.zip › Zorbas et al 2024_Source data_FIG 6/Ataxin3 image1 Phase zoom.tif]

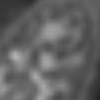

Supplement: Supplementary file 9 — Source data Fig. 6 [file 44319_2024_134_MOESM9_ESM.zip › Zorbas et al 2024_Source data_FIG 6/mHTT image2 Phase zoom.tif]

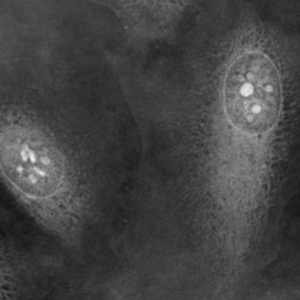

Supplement: Supplementary file 9 — Source data Fig. 6 [file 44319_2024_134_MOESM9_ESM.zip › Zorbas et al 2024_Source data_FIG 6/Ataxin3 image1 Phase.tif]

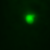

Supplement: Supplementary file 9 — Source data Fig. 6 [file 44319_2024_134_MOESM9_ESM.zip › Zorbas et al 2024_Source data_FIG 6/Ataxin3 image1 GFP zoom.tif]

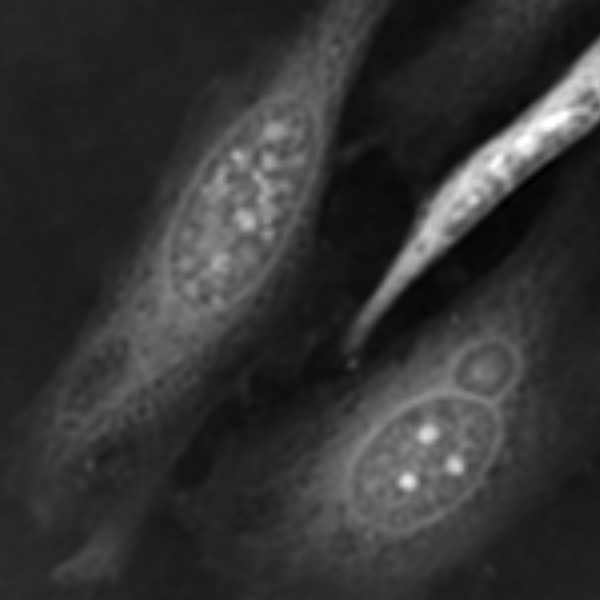

Supplement: Supplementary file 9 — Source data Fig. 6 [file 44319_2024_134_MOESM9_ESM.zip › Zorbas et al 2024_Source data_FIG 6/mHTT image2 Phase.tif]

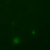

Supplement: Supplementary file 9 — Source data Fig. 6 [file 44319_2024_134_MOESM9_ESM.zip › Zorbas et al 2024_Source data_FIG 6/TDP43 GFP zoom.tif]

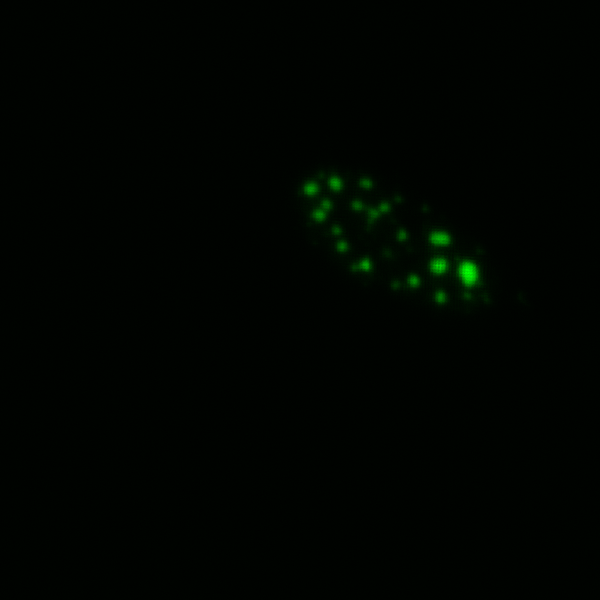

Supplement: Supplementary file 9 — Source data Fig. 6 [file 44319_2024_134_MOESM9_ESM.zip › Zorbas et al 2024_Source data_FIG 6/mHTT image2 GFP.tif]

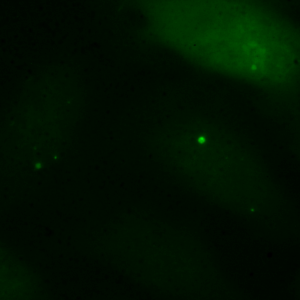

Supplement: Supplementary file 9 — Source data Fig. 6 [file 44319_2024_134_MOESM9_ESM.zip › Zorbas et al 2024_Source data_FIG 6/TDP43 GFP.tif]

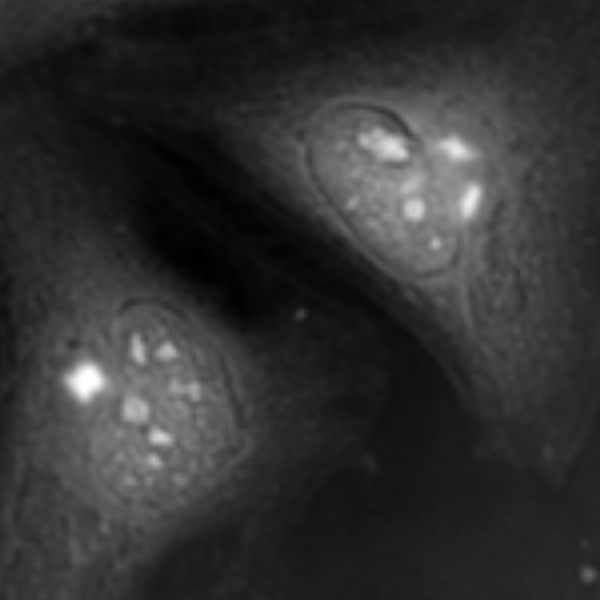

Supplement: Supplementary file 9 — Source data Fig. 6 [file 44319_2024_134_MOESM9_ESM.zip › Zorbas et al 2024_Source data_FIG 6/mHTT image1 Phase.tif]

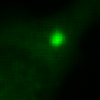

Supplement: Supplementary file 9 — Source data Fig. 6 [file 44319_2024_134_MOESM9_ESM.zip › Zorbas et al 2024_Source data_FIG 6/Ataxin3 image2 GFP zoom.tif]

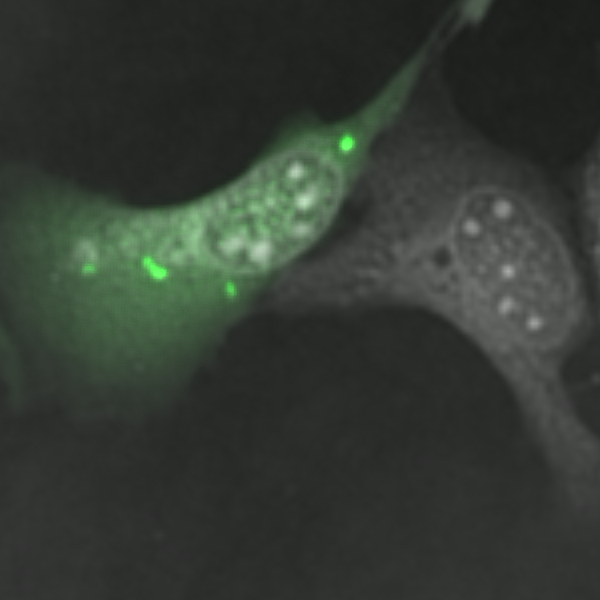

Supplement: Supplementary file 9 — Source data Fig. 6 [file 44319_2024_134_MOESM9_ESM.zip › Zorbas et al 2024_Source data_FIG 6/Ataxin3 image2 overlay.tif]

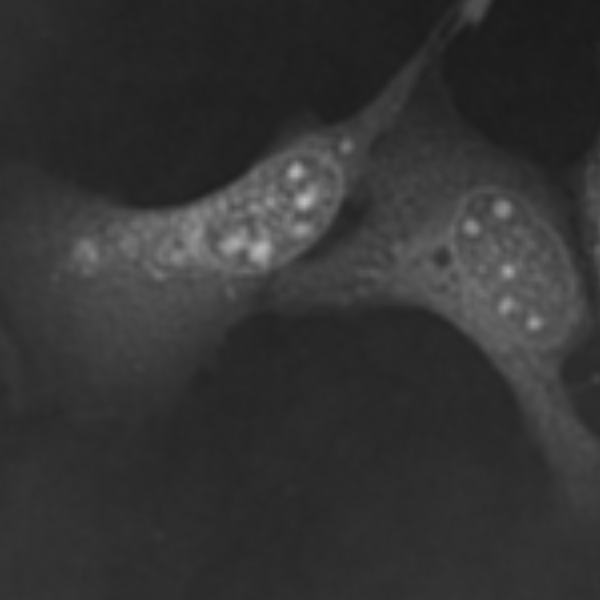

Supplement: Supplementary file 9 — Source data Fig. 6 [file 44319_2024_134_MOESM9_ESM.zip › Zorbas et al 2024_Source data_FIG 6/Ataxin3 image2 Phase.tif]

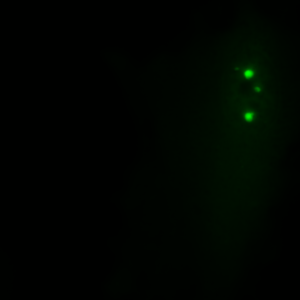

Supplement: Supplementary file 9 — Source data Fig. 6 [file 44319_2024_134_MOESM9_ESM.zip › Zorbas et al 2024_Source data_FIG 6/Ataxin3 image1 GFP.tif]

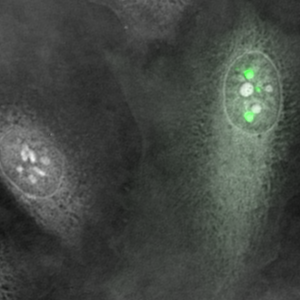

Supplement: Supplementary file 9 — Source data Fig. 6 [file 44319_2024_134_MOESM9_ESM.zip › Zorbas et al 2024_Source data_FIG 6/Ataxin3 image1 overlay.tif]

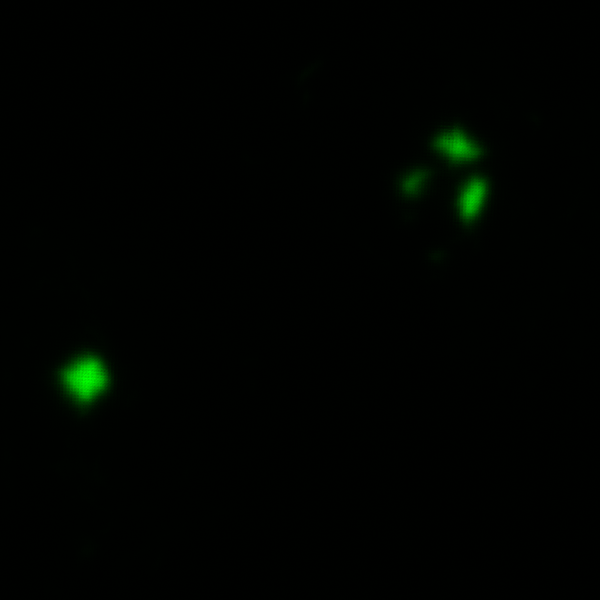

Supplement: Supplementary file 9 — Source data Fig. 6 [file 44319_2024_134_MOESM9_ESM.zip › Zorbas et al 2024_Source data_FIG 6/mHTT image1 GFP.tif]

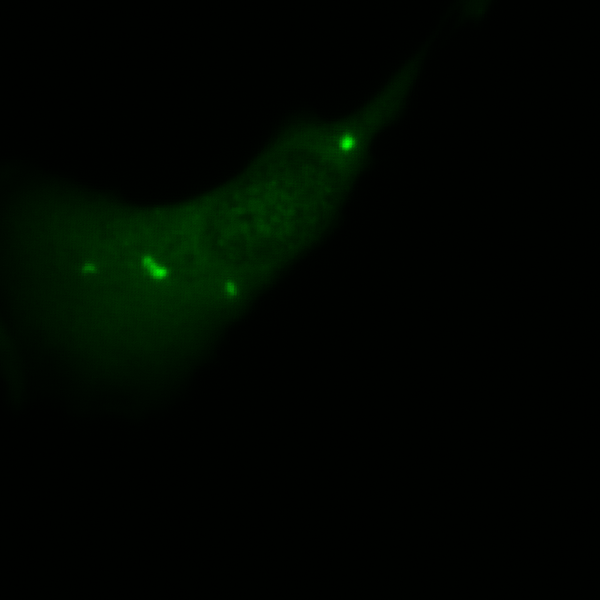

Supplement: Supplementary file 9 — Source data Fig. 6 [file 44319_2024_134_MOESM9_ESM.zip › Zorbas et al 2024_Source data_FIG 6/Ataxin3 image2 GFP.tif]

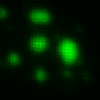

Supplement: Supplementary file 9 — Source data Fig. 6 [file 44319_2024_134_MOESM9_ESM.zip › Zorbas et al 2024_Source data_FIG 6/mHTT image2 GFP zoom.tif]

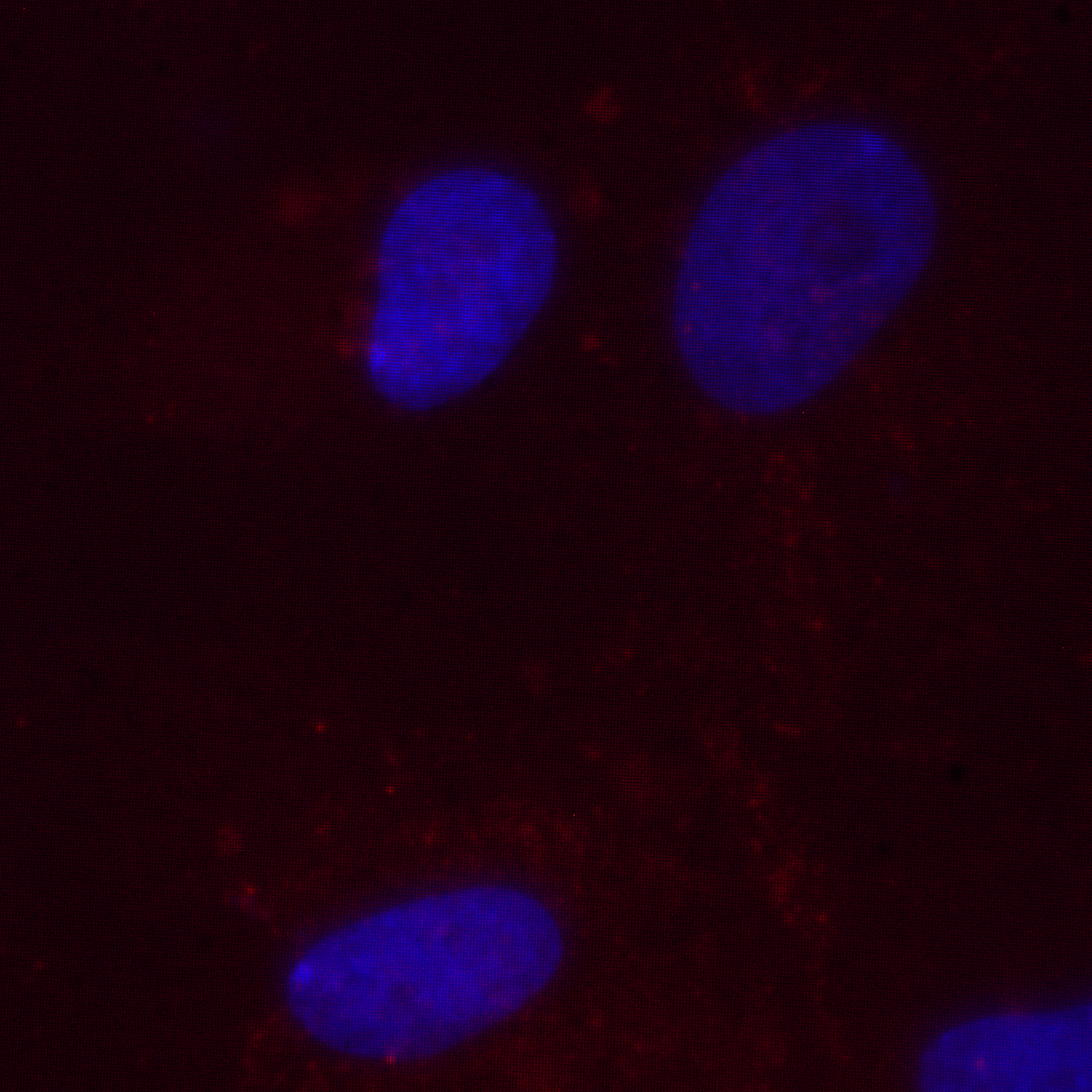

Supplement: Supplementary file 10 — Source data Fig. 7 [file 44319_2024_134_MOESM10_ESM.zip › Zorbas et al 2024_Source data_FIG 7/FIG7A/PRO.tif]

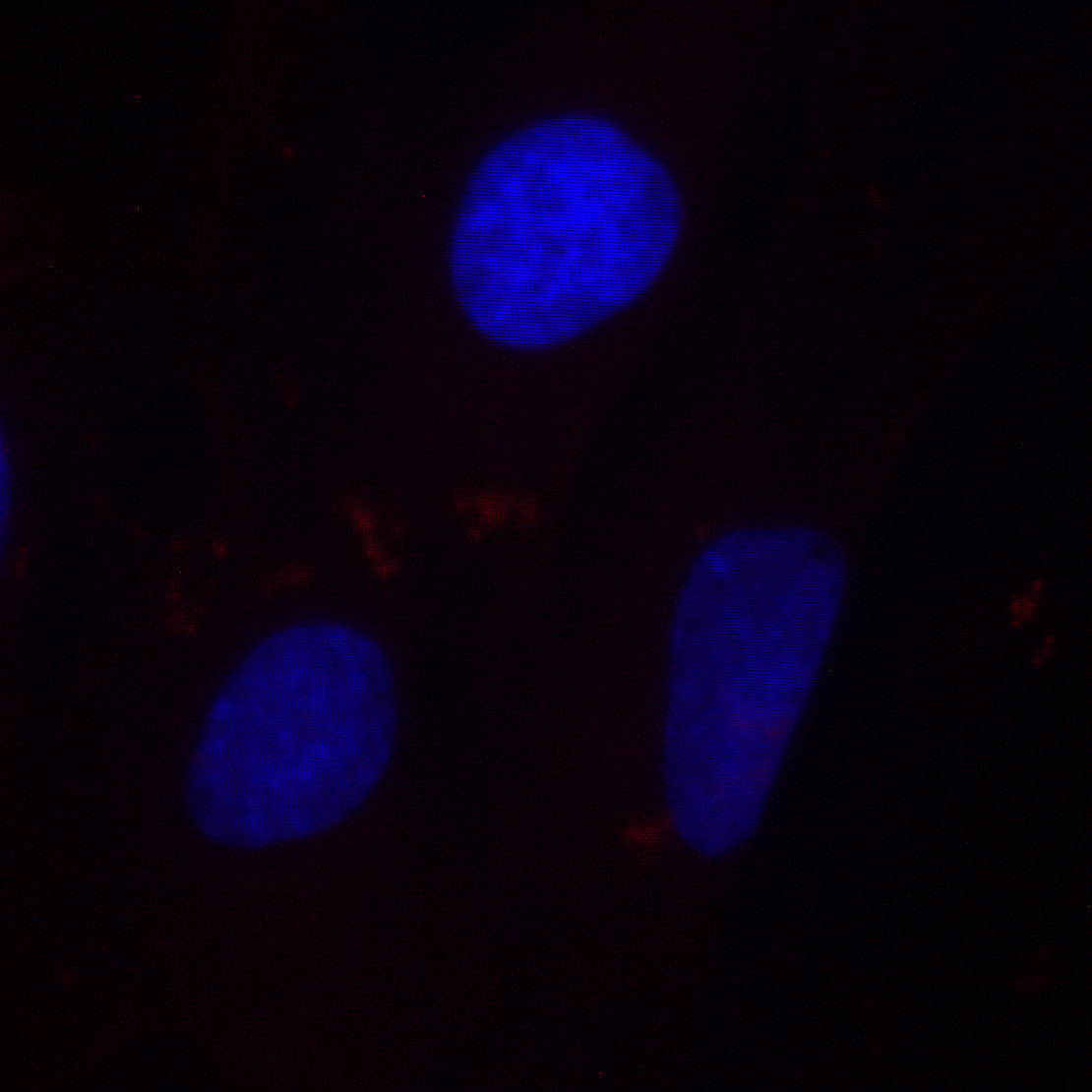

Supplement: Supplementary file 10 — Source data Fig. 7 [file 44319_2024_134_MOESM10_ESM.zip › Zorbas et al 2024_Source data_FIG 7/FIG7A/D0.tif]

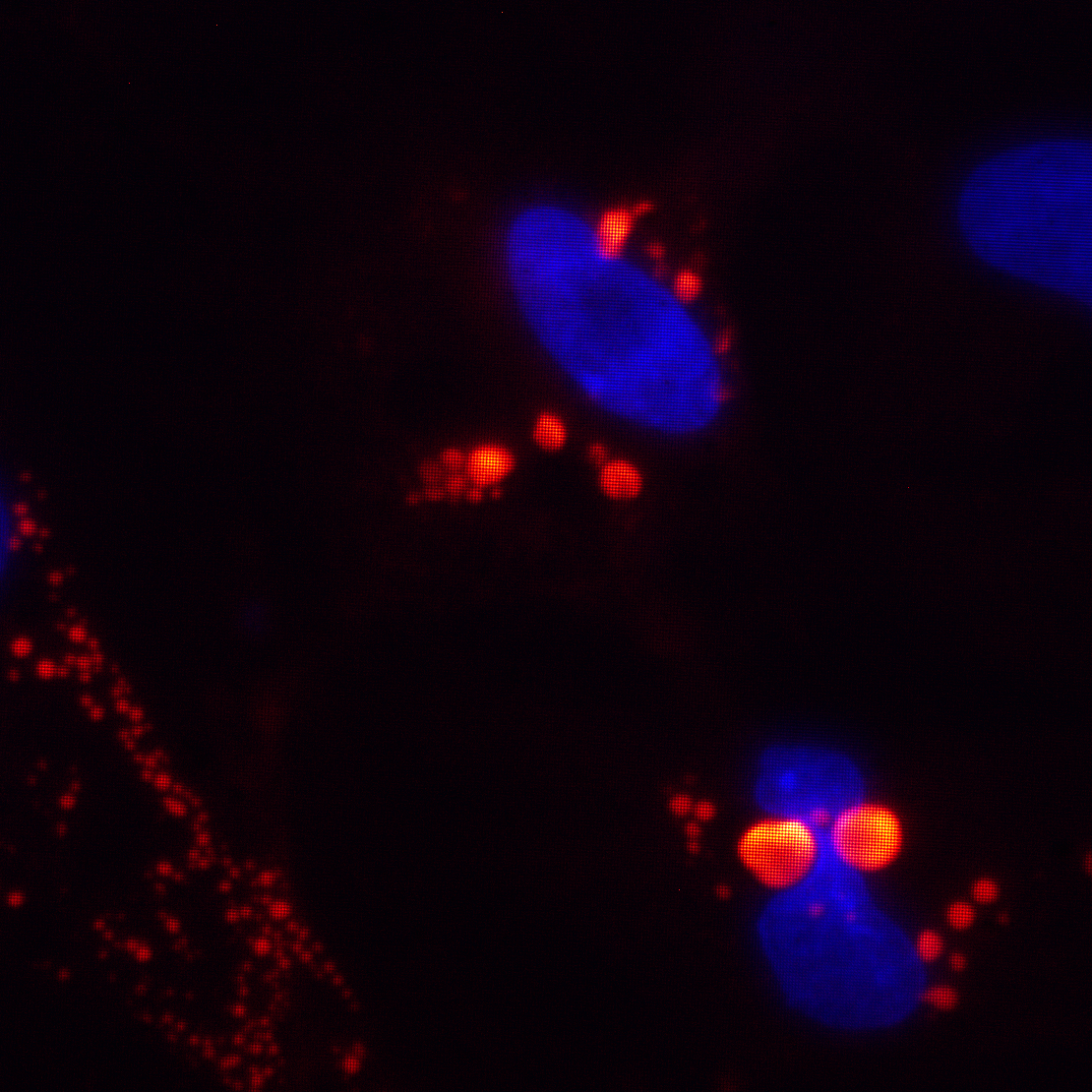

Supplement: Supplementary file 10 — Source data Fig. 7 [file 44319_2024_134_MOESM10_ESM.zip › Zorbas et al 2024_Source data_FIG 7/FIG7A/D12.tif]

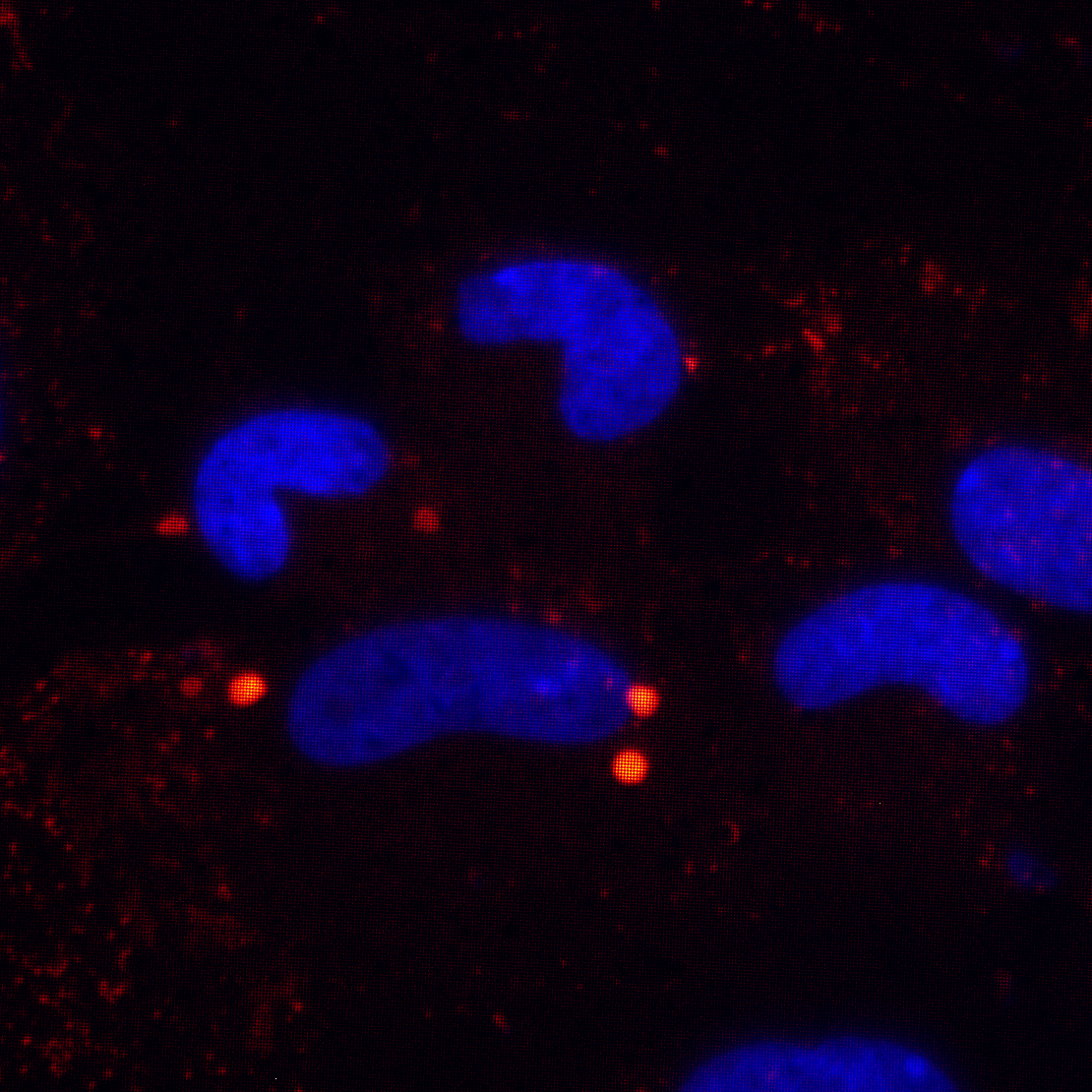

Supplement: Supplementary file 10 — Source data Fig. 7 [file 44319_2024_134_MOESM10_ESM.zip › Zorbas et al 2024_Source data_FIG 7/FIG7A/D6.tif]
