# Supplementary material for: Detecting material state changes in the nucleolus by label-free digital holographic microscopy
Source: EMBO Rep. 2024 Apr 23;25(6):2786–811. doi: 10.1038/s44319-024-00134-5 (PMC11169520; doi:10.1038/s44319-024-00134-5)
Supplement: Supplementary file 6 — Source data Fig. 3 [file 44319_2024_134_MOESM6_ESM.zip › Zorbas et al 2024_Source data_FIG 3/FIG3D/READ ME.docx]

READ ME

Samples were loaded on two replicate gels.

Gel 1 was used to generate blot 1. Blot 1 was hybridized with anti-ul5 (FIG3_D_uL5.tif) and then anti-actin (FIG3_D_Actin.tif) with no stripping in between.

Gel 2 was used to generate blot 2. Blot 2 was hybridized with anti-uL18 (FIG3_D_uL18.tif) and then anti-actin (Actin_replicate_after_ul18_hybridization.tif) with no stripping in between. The molecular weight ladder and both actin and uL18 signals appear on Actin_replicate_after_ul18_hybridization.tif
