# Supplementary figures and images for: Detecting material state changes in the nucleolus by label-free digital holographic microscopy (part 2 of 2)
Source: EMBO Rep. 2024 Apr 23;25(6):2786–811. doi: 10.1038/s44319-024-00134-5 (PMC11169520; doi:10.1038/s44319-024-00134-5)

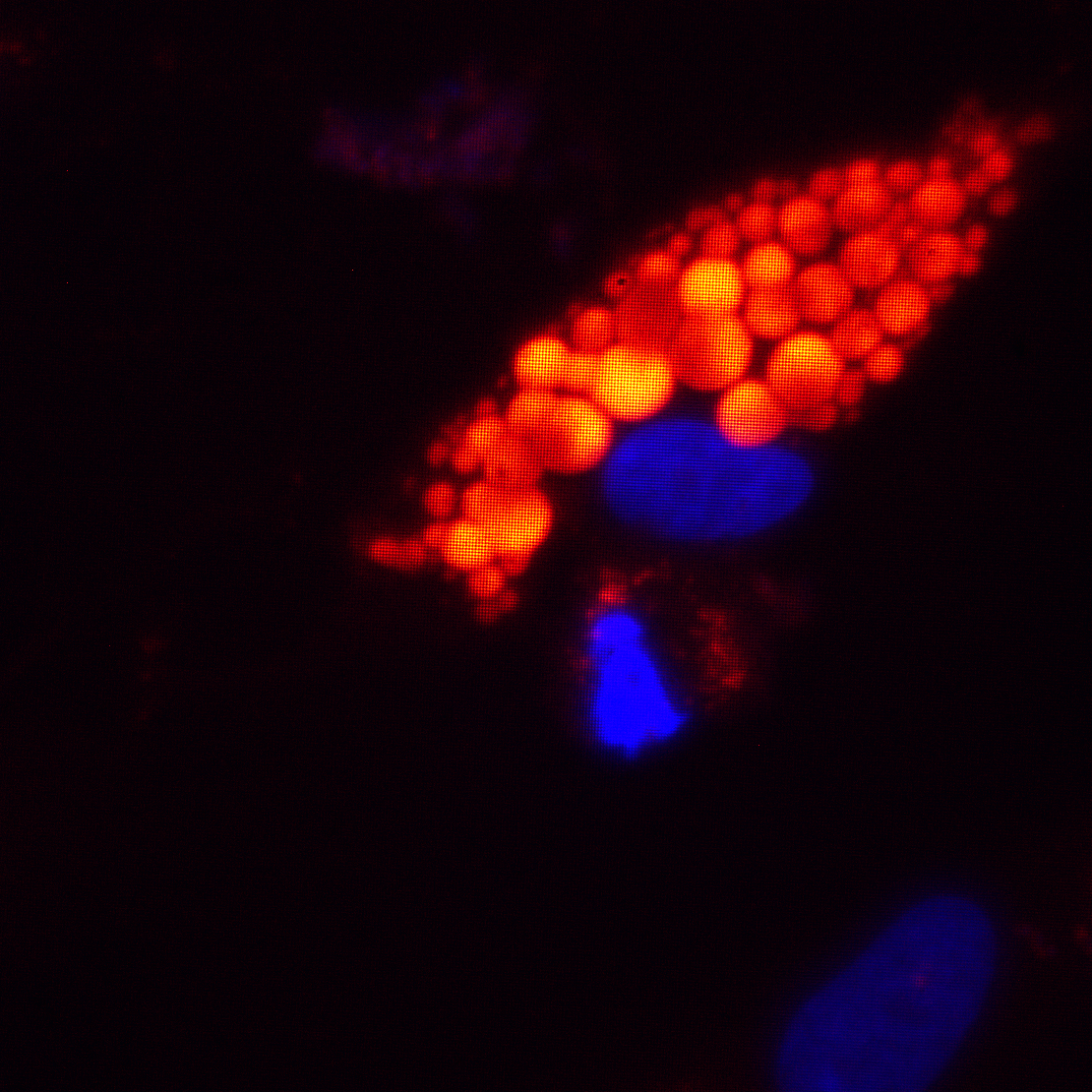

Supplement: Supplementary file 10 — Source data Fig. 7 [file 44319_2024_134_MOESM10_ESM.zip › Zorbas et al 2024_Source data_FIG 7/FIG7A/D15.tif]

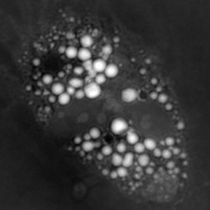

Supplement: Supplementary file 10 — Source data Fig. 7 [file 44319_2024_134_MOESM10_ESM.zip › Zorbas et al 2024_Source data_FIG 7/FIG7B/DHM_D12.tif]

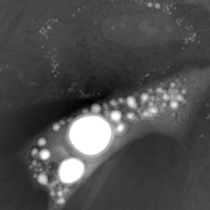

Supplement: Supplementary file 10 — Source data Fig. 7 [file 44319_2024_134_MOESM10_ESM.zip › Zorbas et al 2024_Source data_FIG 7/FIG7B/DHM_D15.tif]

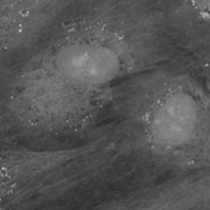

Supplement: Supplementary file 10 — Source data Fig. 7 [file 44319_2024_134_MOESM10_ESM.zip › Zorbas et al 2024_Source data_FIG 7/FIG7B/DHM_undifferentiated.tif]

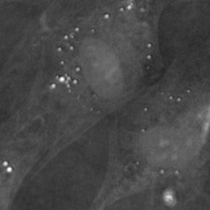

Supplement: Supplementary file 10 — Source data Fig. 7 [file 44319_2024_134_MOESM10_ESM.zip › Zorbas et al 2024_Source data_FIG 7/FIG7B/DHM_D6.tif]

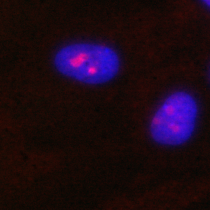

Supplement: Supplementary file 10 — Source data Fig. 7 [file 44319_2024_134_MOESM10_ESM.zip › Zorbas et al 2024_Source data_FIG 7/FIG7B/DAPI_PES1_PLIN1_undifferentiated.tif]

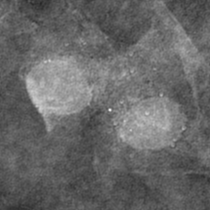

Supplement: Supplementary file 10 — Source data Fig. 7 [file 44319_2024_134_MOESM10_ESM.zip › Zorbas et al 2024_Source data_FIG 7/FIG7B/DHM_D0.tif]

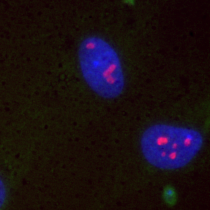

Supplement: Supplementary file 10 — Source data Fig. 7 [file 44319_2024_134_MOESM10_ESM.zip › Zorbas et al 2024_Source data_FIG 7/FIG7B/DAPI_PES1_PLIN1_D6.tif]

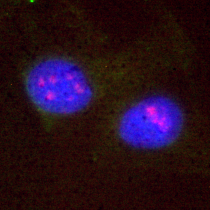

Supplement: Supplementary file 10 — Source data Fig. 7 [file 44319_2024_134_MOESM10_ESM.zip › Zorbas et al 2024_Source data_FIG 7/FIG7B/DAPI_PES1_PLIN1_D0.tif]

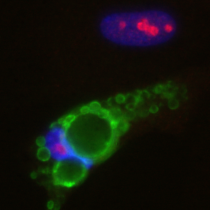

Supplement: Supplementary file 10 — Source data Fig. 7 [file 44319_2024_134_MOESM10_ESM.zip › Zorbas et al 2024_Source data_FIG 7/FIG7B/DAPI_PES1_PLIN1_D15.tif]

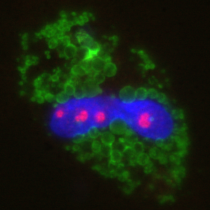

Supplement: Supplementary file 10 — Source data Fig. 7 [file 44319_2024_134_MOESM10_ESM.zip › Zorbas et al 2024_Source data_FIG 7/FIG7B/DAPI_PES1_PLIN1_D12.tif]

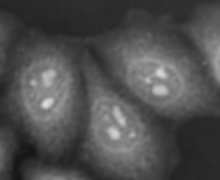

Supplement: Supplementary file 11 — Figure EV1, EV2, EV3, EV4 Source Data [file 44319_2024_134_MOESM11_ESM.zip › Zorbas et al_Source data_FIGS EV1-EV2-EV3-EV4/Zorbas et al 2024_FIG EV3/DMSO Phase.tif]

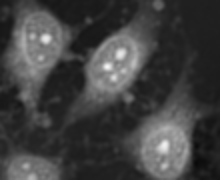

Supplement: Supplementary file 11 — Figure EV1, EV2, EV3, EV4 Source Data [file 44319_2024_134_MOESM11_ESM.zip › Zorbas et al_Source data_FIGS EV1-EV2-EV3-EV4/Zorbas et al 2024_FIG EV3/Latrunculin A Phase.tif]

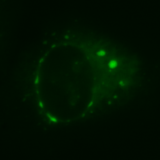

Supplement: Supplementary file 11 — Figure EV1, EV2, EV3, EV4 Source Data [file 44319_2024_134_MOESM11_ESM.zip › Zorbas et al_Source data_FIGS EV1-EV2-EV3-EV4/Zorbas et al 2024_FIG EV4/G3BP-GFP.tif]

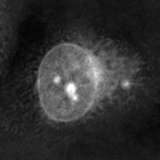

Supplement: Supplementary file 11 — Figure EV1, EV2, EV3, EV4 Source Data [file 44319_2024_134_MOESM11_ESM.zip › Zorbas et al_Source data_FIGS EV1-EV2-EV3-EV4/Zorbas et al 2024_FIG EV4/DHM.tif]

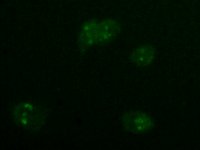

Supplement: Supplementary file 11 — Figure EV1, EV2, EV3, EV4 Source Data [file 44319_2024_134_MOESM11_ESM.zip › Zorbas et al_Source data_FIGS EV1-EV2-EV3-EV4/Zorbas et al 2024_FIG EV2/FIG EV2B/LCL line #1 PES1.tif]

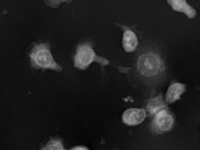

Supplement: Supplementary file 11 — Figure EV1, EV2, EV3, EV4 Source Data [file 44319_2024_134_MOESM11_ESM.zip › Zorbas et al_Source data_FIGS EV1-EV2-EV3-EV4/Zorbas et al 2024_FIG EV2/FIG EV2B/LCL line #2 Phase.tif]

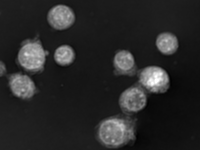

Supplement: Supplementary file 11 — Figure EV1, EV2, EV3, EV4 Source Data [file 44319_2024_134_MOESM11_ESM.zip › Zorbas et al_Source data_FIGS EV1-EV2-EV3-EV4/Zorbas et al 2024_FIG EV2/FIG EV2B/K562 Phase.tif]

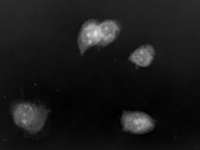

Supplement: Supplementary file 11 — Figure EV1, EV2, EV3, EV4 Source Data [file 44319_2024_134_MOESM11_ESM.zip › Zorbas et al_Source data_FIGS EV1-EV2-EV3-EV4/Zorbas et al 2024_FIG EV2/FIG EV2B/LCL line #1 Phase.tif]

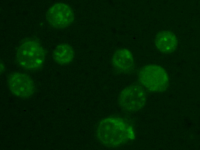

Supplement: Supplementary file 11 — Figure EV1, EV2, EV3, EV4 Source Data [file 44319_2024_134_MOESM11_ESM.zip › Zorbas et al_Source data_FIGS EV1-EV2-EV3-EV4/Zorbas et al 2024_FIG EV2/FIG EV2B/K562 PES1.tif]

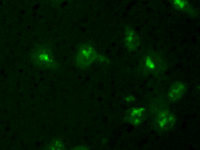

Supplement: Supplementary file 11 — Figure EV1, EV2, EV3, EV4 Source Data [file 44319_2024_134_MOESM11_ESM.zip › Zorbas et al_Source data_FIGS EV1-EV2-EV3-EV4/Zorbas et al 2024_FIG EV2/FIG EV2B/LCL line #2 PES1.tif]

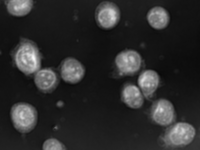

Supplement: Supplementary file 11 — Figure EV1, EV2, EV3, EV4 Source Data [file 44319_2024_134_MOESM11_ESM.zip › Zorbas et al_Source data_FIGS EV1-EV2-EV3-EV4/Zorbas et al 2024_FIG EV2/FIG EV2B/Jurkat Phase.tif]

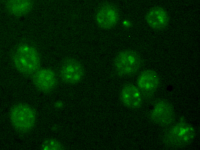

Supplement: Supplementary file 11 — Figure EV1, EV2, EV3, EV4 Source Data [file 44319_2024_134_MOESM11_ESM.zip › Zorbas et al_Source data_FIGS EV1-EV2-EV3-EV4/Zorbas et al 2024_FIG EV2/FIG EV2B/Jurkat PES1.tif]

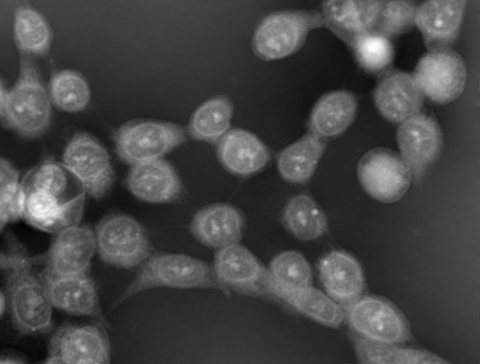

Supplement: Supplementary file 11 — Figure EV1, EV2, EV3, EV4 Source Data [file 44319_2024_134_MOESM11_ESM.zip › Zorbas et al_Source data_FIGS EV1-EV2-EV3-EV4/Zorbas et al 2024_FIG EV2/FIG EV2A/HCT116/HCT116_Phase.tif]

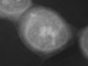

Supplement: Supplementary file 11 — Figure EV1, EV2, EV3, EV4 Source Data [file 44319_2024_134_MOESM11_ESM.zip › Zorbas et al_Source data_FIGS EV1-EV2-EV3-EV4/Zorbas et al 2024_FIG EV2/FIG EV2A/HCT116/HCT116_phase_inset.tif]

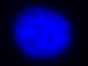

Supplement: Supplementary file 11 — Figure EV1, EV2, EV3, EV4 Source Data [file 44319_2024_134_MOESM11_ESM.zip › Zorbas et al_Source data_FIGS EV1-EV2-EV3-EV4/Zorbas et al 2024_FIG EV2/FIG EV2A/U2OS/U20S_DAPI_inset.png]

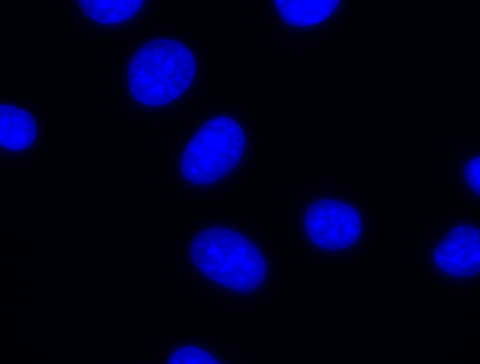

Supplement: Supplementary file 11 — Figure EV1, EV2, EV3, EV4 Source Data [file 44319_2024_134_MOESM11_ESM.zip › Zorbas et al_Source data_FIGS EV1-EV2-EV3-EV4/Zorbas et al 2024_FIG EV2/FIG EV2A/U2OS/U20S_DAPI.tif]

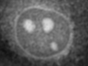

Supplement: Supplementary file 11 — Figure EV1, EV2, EV3, EV4 Source Data [file 44319_2024_134_MOESM11_ESM.zip › Zorbas et al_Source data_FIGS EV1-EV2-EV3-EV4/Zorbas et al 2024_FIG EV2/FIG EV2A/U2OS/U20S_phase_inset.tif]

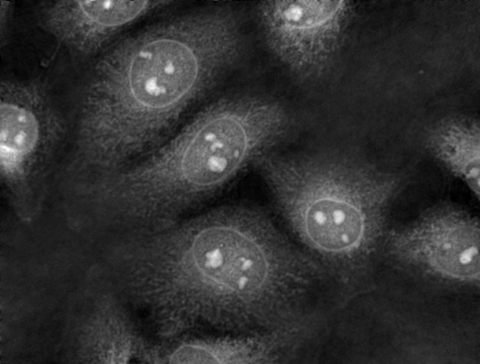

Supplement: Supplementary file 11 — Figure EV1, EV2, EV3, EV4 Source Data [file 44319_2024_134_MOESM11_ESM.zip › Zorbas et al_Source data_FIGS EV1-EV2-EV3-EV4/Zorbas et al 2024_FIG EV2/FIG EV2A/U2OS/U20S_phase.tif]

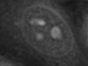

Supplement: Supplementary file 11 — Figure EV1, EV2, EV3, EV4 Source Data [file 44319_2024_134_MOESM11_ESM.zip › Zorbas et al_Source data_FIGS EV1-EV2-EV3-EV4/Zorbas et al 2024_FIG EV2/FIG EV2A/HeLa FBL-GFP/HeLa_FBL-GFP_phase_inset.tif]

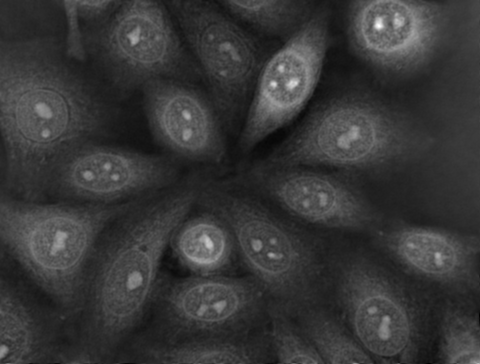

Supplement: Supplementary file 11 — Figure EV1, EV2, EV3, EV4 Source Data [file 44319_2024_134_MOESM11_ESM.zip › Zorbas et al_Source data_FIGS EV1-EV2-EV3-EV4/Zorbas et al 2024_FIG EV2/FIG EV2A/HeLa FBL-GFP/HeLa_FBL-GFP_Phase.tif]

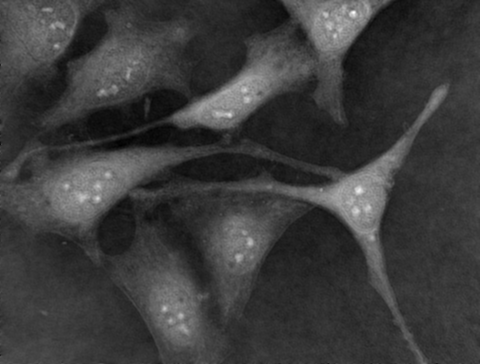

Supplement: Supplementary file 11 — Figure EV1, EV2, EV3, EV4 Source Data [file 44319_2024_134_MOESM11_ESM.zip › Zorbas et al_Source data_FIGS EV1-EV2-EV3-EV4/Zorbas et al 2024_FIG EV2/FIG EV2A/HeLa/HeLa_phase.tif]

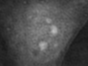

Supplement: Supplementary file 11 — Figure EV1, EV2, EV3, EV4 Source Data [file 44319_2024_134_MOESM11_ESM.zip › Zorbas et al_Source data_FIGS EV1-EV2-EV3-EV4/Zorbas et al 2024_FIG EV2/FIG EV2A/HeLa/HeLa_phase_inset.tif]

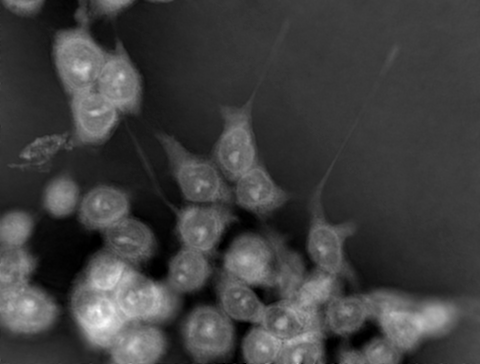

Supplement: Supplementary file 11 — Figure EV1, EV2, EV3, EV4 Source Data [file 44319_2024_134_MOESM11_ESM.zip › Zorbas et al_Source data_FIGS EV1-EV2-EV3-EV4/Zorbas et al 2024_FIG EV2/FIG EV2A/HCT116 FBL-GFP/HCT116_FBL-GFP_phase.tif]

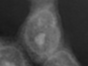

Supplement: Supplementary file 11 — Figure EV1, EV2, EV3, EV4 Source Data [file 44319_2024_134_MOESM11_ESM.zip › Zorbas et al_Source data_FIGS EV1-EV2-EV3-EV4/Zorbas et al 2024_FIG EV2/FIG EV2A/HCT116 FBL-GFP/HCT116_FBL-GFP_phase_inset.tif]

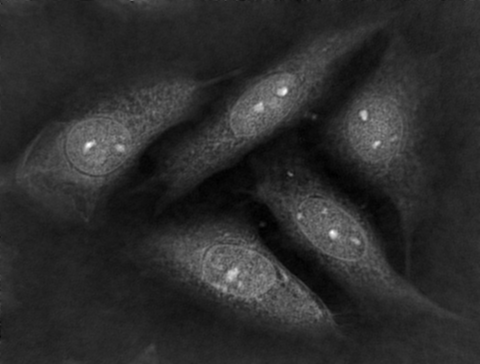

Supplement: Supplementary file 11 — Figure EV1, EV2, EV3, EV4 Source Data [file 44319_2024_134_MOESM11_ESM.zip › Zorbas et al_Source data_FIGS EV1-EV2-EV3-EV4/Zorbas et al 2024_FIG EV2/FIG EV2A/SiHa/SiHa_phase.tif]

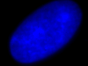

Supplement: Supplementary file 11 — Figure EV1, EV2, EV3, EV4 Source Data [file 44319_2024_134_MOESM11_ESM.zip › Zorbas et al_Source data_FIGS EV1-EV2-EV3-EV4/Zorbas et al 2024_FIG EV2/FIG EV2A/SiHa/SiHa_DAPI_inset.tif]

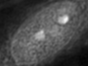

Supplement: Supplementary file 11 — Figure EV1, EV2, EV3, EV4 Source Data [file 44319_2024_134_MOESM11_ESM.zip › Zorbas et al_Source data_FIGS EV1-EV2-EV3-EV4/Zorbas et al 2024_FIG EV2/FIG EV2A/SiHa/SiHa_phase_inset.tif]

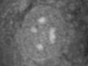

Supplement: Supplementary file 11 — Figure EV1, EV2, EV3, EV4 Source Data [file 44319_2024_134_MOESM11_ESM.zip › Zorbas et al_Source data_FIGS EV1-EV2-EV3-EV4/Zorbas et al 2024_FIG EV2/FIG EV2A/A549/A549_phase_inset.tif]

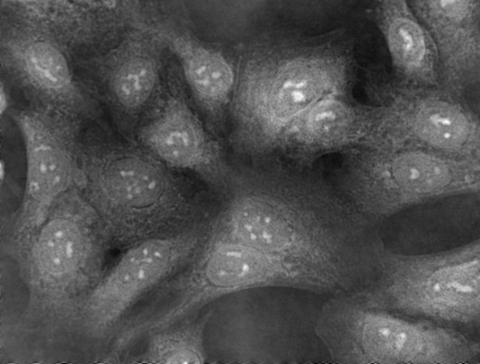

Supplement: Supplementary file 11 — Figure EV1, EV2, EV3, EV4 Source Data [file 44319_2024_134_MOESM11_ESM.zip › Zorbas et al_Source data_FIGS EV1-EV2-EV3-EV4/Zorbas et al 2024_FIG EV2/FIG EV2A/A549/A549_phase.tif]

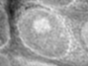

Supplement: Supplementary file 11 — Figure EV1, EV2, EV3, EV4 Source Data [file 44319_2024_134_MOESM11_ESM.zip › Zorbas et al_Source data_FIGS EV1-EV2-EV3-EV4/Zorbas et al 2024_FIG EV2/FIG EV2A/MCF7/MCF7_phase_inset.tif]

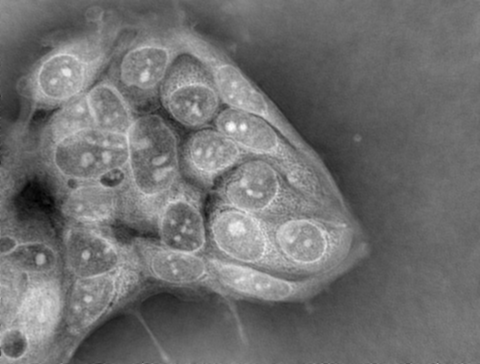

Supplement: Supplementary file 11 — Figure EV1, EV2, EV3, EV4 Source Data [file 44319_2024_134_MOESM11_ESM.zip › Zorbas et al_Source data_FIGS EV1-EV2-EV3-EV4/Zorbas et al 2024_FIG EV2/FIG EV2A/MCF7/MCF7_phase.tif]
