# Supplementary material for: Detecting material state changes in the nucleolus by label-free digital holographic microscopy
Source: EMBO Rep. 2024 Apr 23;25(6):2786–811. doi: 10.1038/s44319-024-00134-5 (PMC11169520; doi:10.1038/s44319-024-00134-5)
Supplement: Supplementary file 13 — Table EV1 [file 44319_2024_134_MOESM13_ESM.docx]

**Table EV1. Quantification of Figure 5**

| **Panel F** | Mean | SD | n | Total number of nucleoli analyzed |
| --- | --- | --- | --- | --- |
| No BL | 78.19 | 17.72 | 10 | 300 |
| Medium BL | 85.58 | 18.66 | 10 | 301 |
| High BL | 103.2 | 11.94 | 10 | 335 |
| Statistics:  One-way ANOVA p=0.0062  Uncorrected Fisher’s LSD results:  No BL vs. Medium BL p=0.322  No BL vs. High BL p=0.002  Medium BL vs. High BL p=0.023 | | | | |
