# Supplementary material for: Detecting material state changes in the nucleolus by label-free digital holographic microscopy
Source: EMBO Rep. 2024 Apr 23;25(6):2786–811. doi: 10.1038/s44319-024-00134-5 (PMC11169520; doi:10.1038/s44319-024-00134-5)
Supplement: Supplementary file 14 — Table EV2 [file 44319_2024_134_MOESM14_ESM.docx]

**Table EV2. Quantification of Figure 3**

| **Panel B, Drugs** | Mean | SD | n | Total number of nucleoli analyzed |
| --- | --- | --- | --- | --- |
| DMSO | 72.09 | 6.54 | 10 | 287 |
| DRB | 54.28 | 4.64 | 10 | 290 |
| ROS | 49.39 | 5.37 | 10 | 256 |
| Actinomycin D | 77.45 | 4.53 | 10 | 234 |
| CX-5461 | 77.70 | 6.58 | 10 | 301 |
| Statistics:  One-way ANOVA p<0.0001  Uncorrected Fisher’s LSD results:  DMSO vs. DRB p<0.0001  DMSO vs. ROS p<0.0001  DMSO vs. AcD p=0.038  DMSO vs. CX-5461 p=0.030 | | | | |

| **Panel E, Factor depletion** | Mean | SD | n | Total number of nucleoli analyzed |
| --- | --- | --- | --- | --- |
| SCR | 61.26 | 7.10 | 11 | 332 |
| uL5 | 45.49 | 5.34 | 11 | 311 |
| uL18 | 49.15 | 4.29 | 11 | 235 |
| Statistics:  One-way ANOVA p<0.0001  Uncorrected Fisher’s LSD results:  SCR vs. uL5 p<0.0001  SCR vs. uL18 p<0.0001 | | | | |
