# Supplementary material for: Detecting material state changes in the nucleolus by label-free digital holographic microscopy
Source: EMBO Rep. 2024 Apr 23;25(6):2786–811. doi: 10.1038/s44319-024-00134-5 (PMC11169520; doi:10.1038/s44319-024-00134-5)
Supplement: Supplementary file 15 — Table EV3 [file 44319_2024_134_MOESM15_ESM.docx]

**Table EV3. Quantification of Figure EV3**

| **Nucleolus** | Mean | SD | n | Total number of nucleoli analyzed |
| --- | --- | --- | --- | --- |
| DMSO | 74.55 | 2.59 | 5 | 196 |
| Latrunculin A | 123.7 | 8.35 | 5 | 257 |
| Statistics:  Unpaired t-test p<0.0001 | | | | |

| **Cytoplasm** | Mean | SD | n | Total number of cells analyzed |
| --- | --- | --- | --- | --- |
| DMSO | 47.82 | 13.70 | 5 | 137 |
| Latrunculin A | 34.30 | 13.41 | 5 | 129 |
| Statistics:  Unpaired t-test p=0.1535 | | | | |

| **Nucleolar circularity** | Mean | SD | n | Total number of nucleoli analyzed |
| --- | --- | --- | --- | --- |
| DMSO | 0.613 | 0.035 | 5 | 461 |
| Latrunculin A | 0.689 | 0.034 | 5 | 649 |
| Statistics:  Unpaired t-test p=0.009 | | | | |

| **Nucleolar area** | Mean | SD | n | Total number of nucleoli analyzed |
| --- | --- | --- | --- | --- |
| DMSO | 9.967 | 1.073 | 5 | 461 |
| Latrunculin A | 7.090 | 0.789 | 5 | 649 |
| Statistics:  Unpaired t-test p=0.0013 | | | | |
