# Supplementary material for: Detecting material state changes in the nucleolus by label-free digital holographic microscopy
Source: EMBO Rep. 2024 Apr 23;25(6):2786–811. doi: 10.1038/s44319-024-00134-5 (PMC11169520; doi:10.1038/s44319-024-00134-5)
Supplement: Supplementary file 16 — Expanded View Figures [file 44319_2024_134_MOESM16_ESM.pdf]

Expanded View Figures

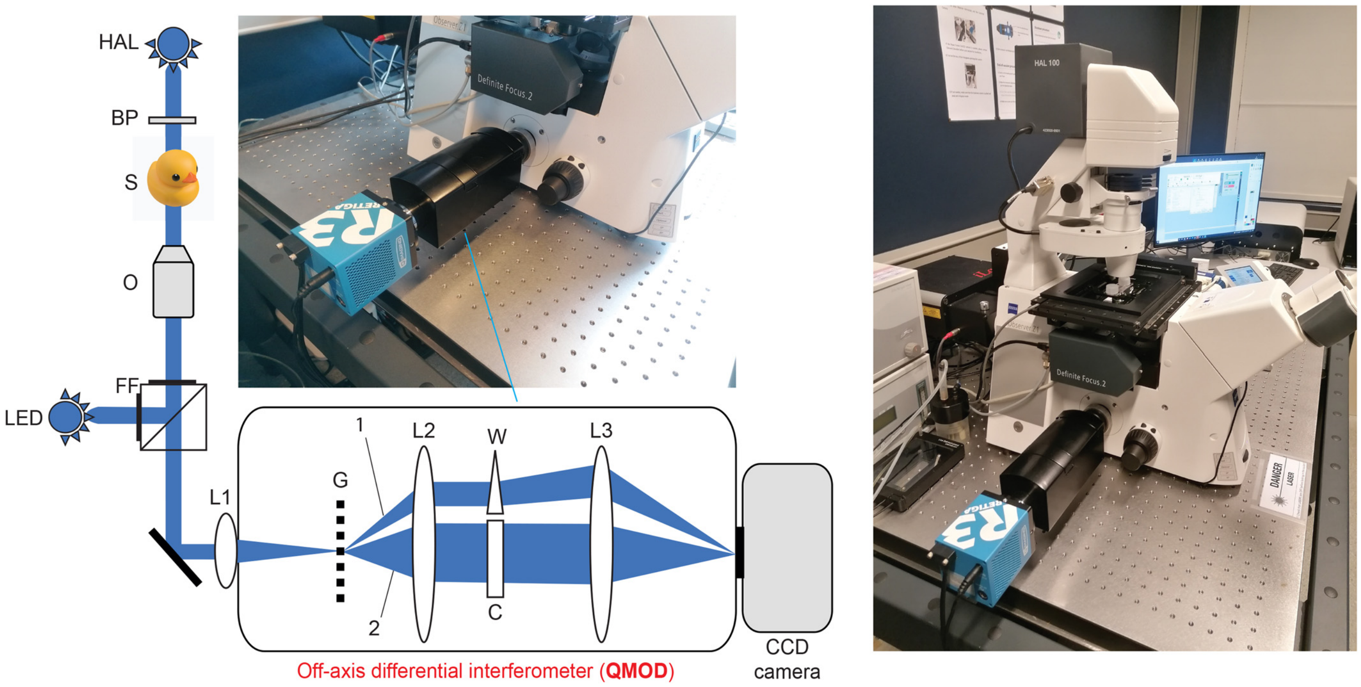

Figure EV1. Off-axis differential interferometer (QMOD) setup used in this work.

Description of the beam path and microscope configuration used. The diagram illustrates the detailed beam path in our QMOD setup. A purposely built versatile "plug-in" DHM adapter (QMOD, developed together with Ovizio s.a.) was connected between the lateral port of a Zeiss inverted microscope and a Retiga R3 camera (Qimaging) used for imaging the DHM phase and all fluorescence channels. The camera was driven from the MetaXpress (Molecular Devices) environment. Images were processed with OsOne (Ovizio). Digital holograms were recorded with an incoherent light source (**HAL** lamp) on an inverted microscope adjusted for proper Köhler illumination and coupled to a QMOD interferometer and CCD camera. A bandpass filter (**BP**, 550 nm) was used to increase the coherence of the light and obtain the partial coherence required for holography. The image-forming light rays passing through the specimen (**S**) were captured with the microscope objective (**O**) and directed from microscope lens **L1** to the QMOD interferometer. In the QMOD, a diffraction grating **G** induced splitting of the incident light beam into a diffracted beam (**1**, reference) and a non-diffracted light beam (**2**, object beam). A second lens (**L2**) placed at the focal distance from the grating G reshaped both the diffracted and non-diffracted beams into beams parallel to the optical axis. A wedge (**W**) inserted in the optical path of the object beam induced a slight shift of the images produced by the diffracted and non-diffracted light beams. **C** is a compensating optical module placed in the optical path of the non-diffracted light beam to compensate for the light shift introduced by W in the diffracted beam. The diffracted beam is then recombined with the object beam and focalized by means of objective lens **L3** on the recording plane of a CCD camera, where the hologram is recorded. LED, illumination; FF, fluorescent filter cube. Fluorescence imaging: excitation illumination is emitted by a light-emitting diode (**LED**) and is directed to the sample (**S**) through a fluorescence filter cube (**FF**). Fluorescence emission by the specimen is collected by the objective, passes through the filter cube and **L1**, enters the QMOD, and finally reaches the CCD camera.

 © The Author(s)

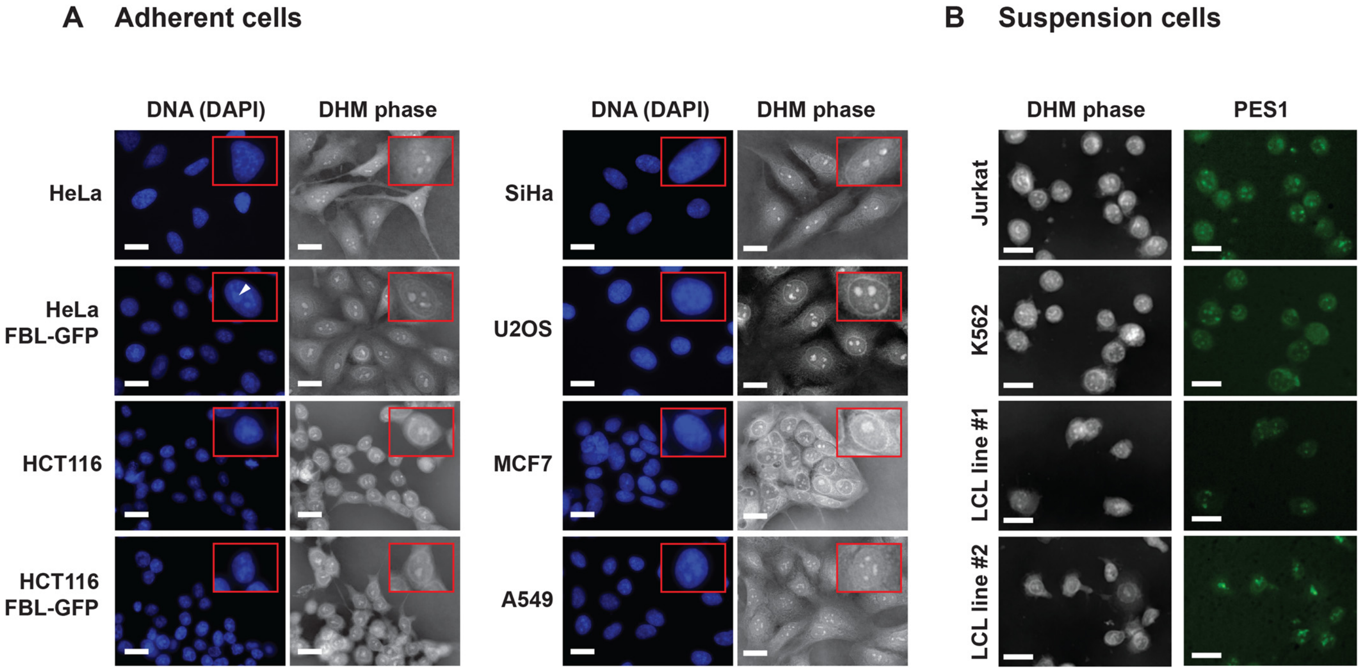

Figure EV2. DHM detection of the nucleolus in cell lines of various origins.

Cells stained with DAPI to detect the DNA-rich nucleoplasm were observed by correlative DHM-fluorescence microscopy. (**A**) Adherent cells: HeLa, HCT116, SiHa, U2OS, MCF7, and A549. Insets, magnification of an individual cell nucleus. An example of a perinucleolar chromatin ring, lining the nucleolus, is highlighted with an arrowhead in the HeLa-FBL-GFP panel for reference (see Fig. 2B for details). Scale bar, 20 μm. (**B**) Suspension cells: Jurkat, K562, and lymphoblastoid cell lines (LCL). LCL #1 is from a healthy individual; LCL #2 is from a patient expressing a mutation in RPL5. Cells were stained with an antibody against PES1 to detect the nucleolus. Source data are available online for this figure.

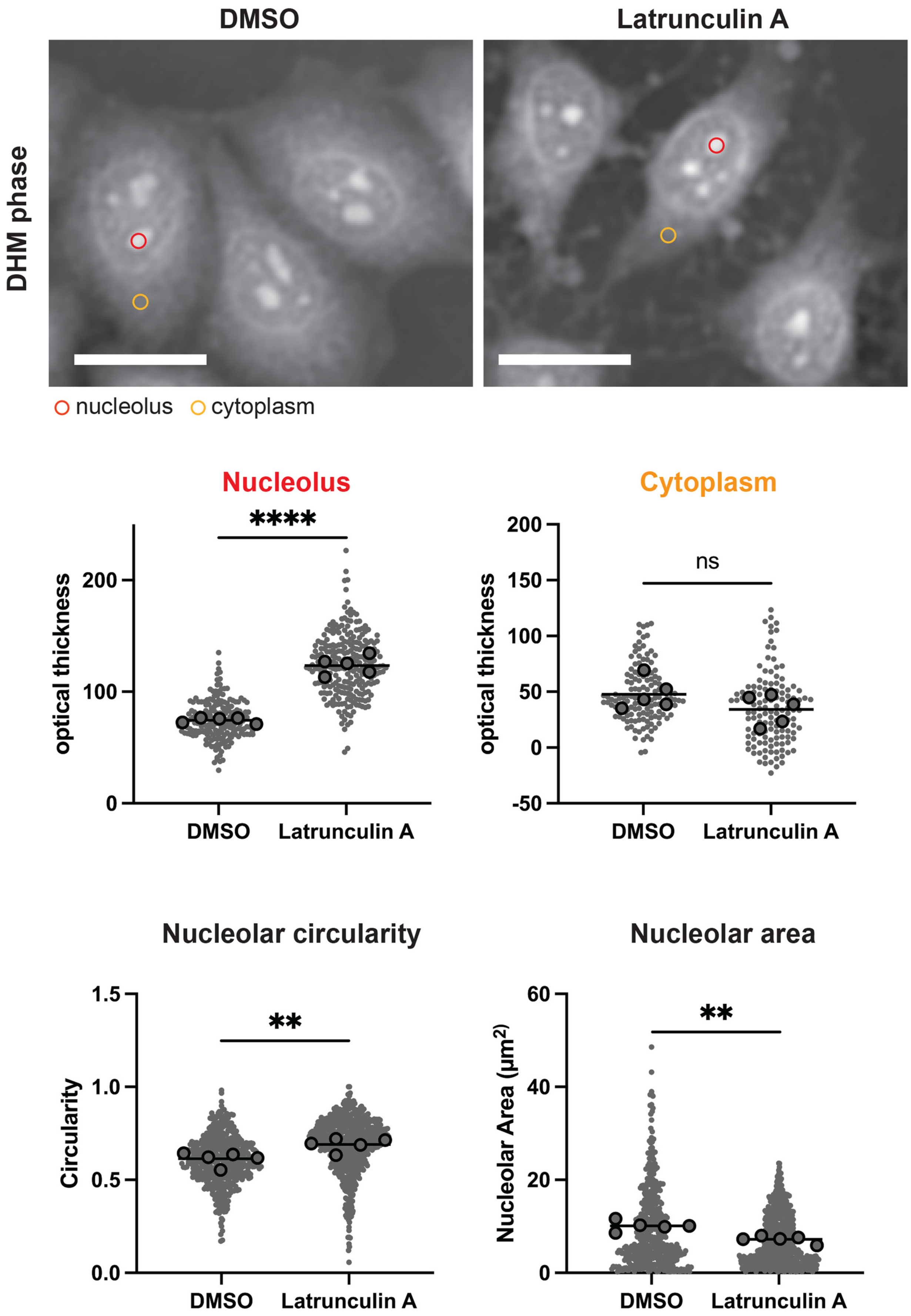
DMSO
Latrunculin A
DHM phase
nucleolus
cytoplasm
Nucleolus
****
optical thickness
200
100
0
DMSO
Latrunculin A
Cytoplasm
ns
200
150
100
50
0
-50
Nucleolar circularity
**
Circularity
1.5
1.0
0.5
0.0
Nucleolar area
**
Nucleolar Area (μm²)
60
40
20
0

 © The Author(s)

Figure EV3. DHM assessment of the effects of latrunculin A on cell material state.

Quantification of the optical thickness of the nucleolus and cytoplasm in cells treated with the actin cytoskeleton depolymerizing drug latrunculin A (500 mM, 30 min). As control, DMSO was used. Latrunculin A leads to a decrease in cytoplasmic optical thickness and an increase in nucleolar optical thickness. Scale bar, 20 µm. Number of independent experiments, $n = 5$. The mean of each independent experiment is represented by a black circle, the mean of the means by a black line, and the individual nucleoli counted by gray dots. The total numbers of nucleoli counted were 196 and 257 for DMSO and latrunculin A, respectively. The total numbers of cells whose cytoplasm was analyzed were 137 and 129, for DMSO and latrunculin A, respectively. Data were analyzed with the unpaired *t*-test ($p < 0.0001$ for the nucleolus; $p = 0.1535$, for the cytoplasm). Additionally, the nucleolar area and circularity were measured. The nucleolar area is significantly reduced (unpaired *t*-test, $p = 0.0013$). The nucleolar circularity, calculated as $4\pi * \text{area}/\text{perimeter}^2$ is also significantly increased upon latrunculin A treatment (unpaired *t*-test, $p = 0.009$). ns, $p > 0.05$; **$p \leq 0.01$; ****$p \leq 0.0001$. Source data are available online for this figure.

© The Author(s)

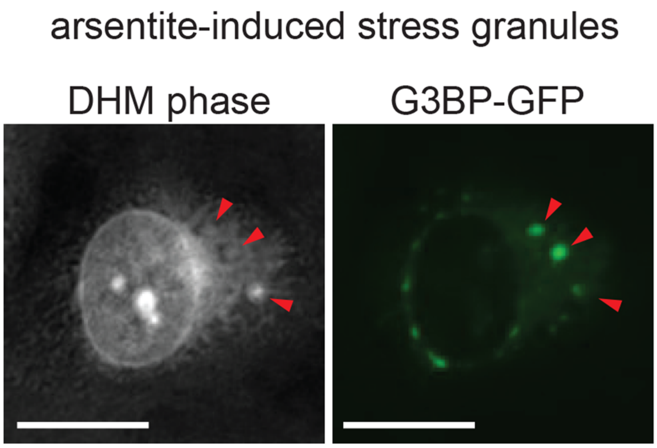

Figure EV4. Cytoplasmic stress granules can be detected by DHM.

Stress granule formation was induced in U2OS cells expressing a G3BP-GFP construct by treating them with arsenite (0.5 mM sodium arsenite, 1 h). G3PB detection in the fluorescence channel allowed monitoring the stress granules (red arrowheads), some of which were also visible in the DHM phase. Scale bar, 20 μm. Source data are available online for this figure.

 © The Author(s)
